# A chair-type G-quadruplex structure formed by a human telomeric variant DNA in K^+^ solution†
†Electronic supplementary information (ESI) available. See DOI: 10.1039/c8sc03813a


**DOI:** 10.1039/c8sc03813a

**Published:** 2018-10-04

**Authors:** Changdong Liu, Bo Zhou, Yanyan Geng, Dick Yan Tam, Rui Feng, Haitao Miao, Naining Xu, Xiao Shi, Yingying You, Yuning Hong, Ben Zhong Tang, Pik Kwan Lo, Vitaly Kuryavyi, Guang Zhu

**Affiliations:** a Division of Life Science , The Hong Kong University of Science and Technology , Clear Water Bay , Kowloon , Hong Kong SAR , China . Email: gzhu@ust.hk; b Institute for Advanced Study , The Hong Kong University of Science and Technology , Clear Water Bay , Kowloon , Hong Kong SAR , China; c Department of Biology and Chemistry , City University of Hong Kong , 83 Tat Chee Avenue , Kowloon Tong , Hong Kong SAR , China; d Department of Chemistry , The Hong Kong University of Science and Technology , Clear Water Bay , Kowloon , Hong Kong SAR , China; e Structural Biology Program , Memorial Sloan-Kettering Cancer Center , New York , NY , USA . Email: v.kuryavyi@gmail.com; f Institute for Advanced Study and State Key Laboratory of Molecular Neuroscience , The Hong Kong University of Science and Technology , Clear Water Bay , Kowloon , Hong Kong SAR , China

## Abstract

Guanine tracts of human telomeric DNA sequences are known to fold into eight different four-stranded structures that vary by the conformation of guanine nucleotides arranged in the stack of G-tetrads in their core and by different kinds and orders of connecting loops, called G-quadruplexes. Here, we present a novel G-quadruplex structure formed in K^+^ solution by a human telomeric variant d[(GGGTTA)2GGGTTTGGG], *htel21*T_18_. This variant DNA is located in the subtelomeric regions of human chromosomes 8, 11, 17, and 19 as well as in the DNase hypersensitive region and in the subcentromeric region of chromosome 5. Interestingly, single A18T substitution that makes *htel21*T_18_ different from the human telomeric sequence results in the formation of a three-layer chair-type G-quadruplex, a fold previously unknown among human telomeric repeats, with two loops interacting through the reverse Watson–Crick A6·T18 base pair. The loops are edgewise; glycosidic conformation of guanines is *syn*·*anti*·*syn*·*anti* around each tetrad, and each strand of the core has two antiparallel adjacent strands. Our results expand the repertoire of known G-quadruplex folding topologies and may provide a potential target for structure-based anticancer drug design.

## Abstract 2

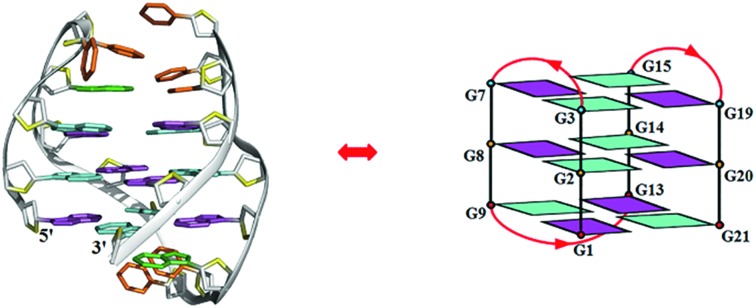
The chair-type G-quadruplex structure formed by human telomeric variant DNA.

## Introduction

Telomeres are highly repetitive DNA regions located at the ends of linear eukaryotic chromosomes. Their function is to protect the terminal ends of chromosomes from being recognized as damaged DNA and to support faithful chromosome replication during each cell cycle.^1^,^2^ Human telomeric DNA contains tandem repeats of the sequence 5′-GGGTTA-3′.^3^ Under physiological ionic conditions, this guanine-rich strand can fold into a variety of four-stranded G-quadruplex structures involving G-tetrads,^4^–^9^ which are important for telomere biology and are currently attractive targets for the development of anti-cancer drugs.^10^–^13^

Many different G-quadruplex topologies are known^4^–^8^ and the four-repeat human telomeric G-rich sequences can adopt a range of intramolecular G-quadruplex structures.^8^,^14^ Eight different unimolecular G-quadruplex structures of various human telomeric DNA sequences containing four canonical GGGTTA repeats have been solved by NMR or X-ray crystallography under different experimental conditions (Fig. S1†).^15^–^20^ All of these structures contain the 21 nt human telomeric sequence d[(GGGTTA)_3_GGG], termed *htel21*, which should be the shortest sequence in length for the formation of an intramolecular G-quadruplex.

We questioned how the sequence variants with single or double nucleotide substitution in the TTA loops found as subtelomeric repeats in human chromosomes affect the G-quadruplex fold. Based on bioinformatics studies and with the use of CD and NMR spectroscopy, we have found a variant telomeric DNA *htel21*T_18_ that has a T substitution at A18 of *htel21* and showed that it adopts a chair-type monomolecular G-quadruplex with three G-tetrad layers which was hitherto unknown among human telomeric quadruplex forms. In this structure, the loop–loop interactions are mediated by the reverse Watson–Crick A6·T18 base pair and, in addition, there is a hydrogen bond between T5 and T16. In the *htel21*T_18_ G-quadruplex the loops are successively edgewise; glycosidic conformation of guanines is *syn*·*anti*·*syn*·*anti* around each tetrad, and each strand of the core has two antiparallel adjacent strands.

Bioinformatics studies have shown localizations of *htel21*T_18_ and its repeats in the subtelomeric regions of human chromosomes 8, 11, 17, and 19 as well as in the subcentromeric region of chromosome 5. Interestingly, the sequence *htel21*T_18_ can also be localized in a DNase hypersensitive region, implying that this chromosome segment has a propensity to form a chair-like G-quadruplex *in vivo*. This novel G-quadruplex form expands the repertoire of known G-quadruplex folding topologies and may provide a potential target for structure-based anticancer drug design.

## Results

A human telomeric variant DNA, *htel21*T_18_, forms a stable intramolecular G-quadruplex structure in K^+^ solution.

We screened the human genome using single *htel21* variant DNAs which contain single A-to-T and T-to-A and double TT-to-AA substitutions at the various thymine and adenine positions as inputs for BLAT search (; http://genome.ucsc.edu/). As shown in Fig S2,† ten 21 nt human telomeric variants were found in the human genome.

**Fig. 1:**
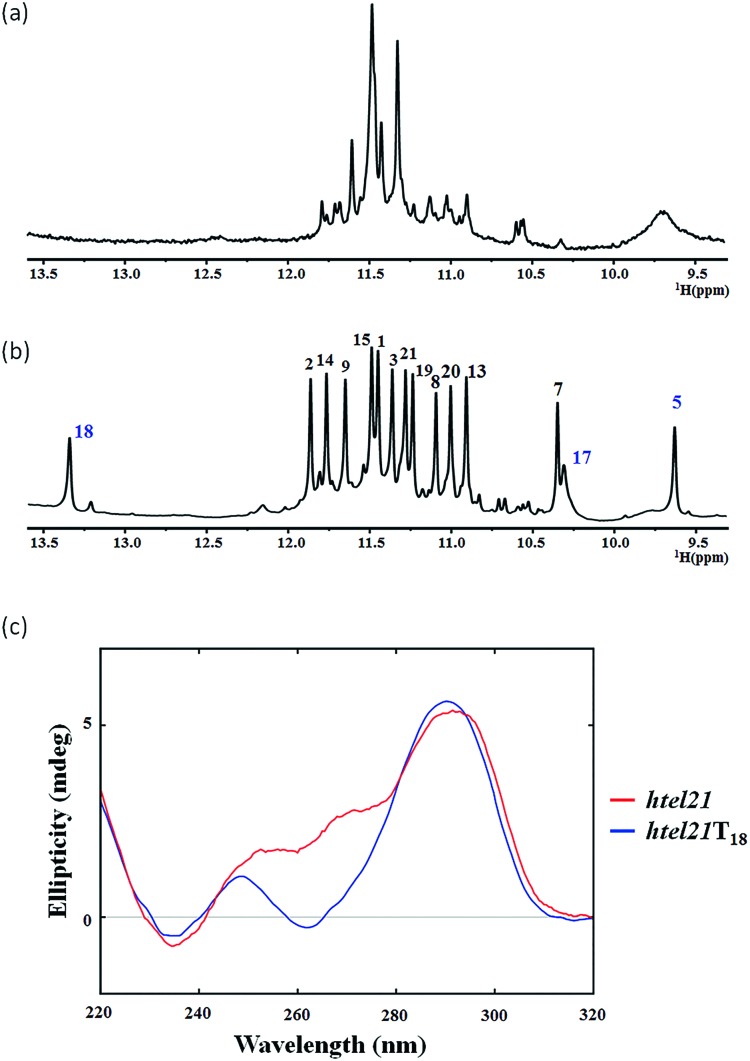
(a) The imino region of the 1D ^1^H-NMR spectrum of the human telomeric d[(GGGTTA)_3_GGG] DNA, *htel21*. (b) The imino region of the 1D ^1^H-NMR spectrum of the human telomeric variant d[(GGGTTA)_2_GGGTT*T*GGG] DNA (*htel21*T_18_) where A18 was replaced with T in K^+^ solution. Both spectra were recorded at 10 °C. (c) CD spectra of *htel21* and *htel21*T_18_ in 70 mM KCl and 20 mM potassium phosphate solution recorded at 25 °C.

The *htel21* appeared to form a mixture of G-quadruplex conformations in the presence of K^+^, as indicated by the 1D ^1^H NMR spectrum (Fig. 1a) and the CD spectrum (Fig. 1c). Surprisingly, we found an *htel21*T_18_ variant (d[(GGGTTA)_2_GGGTT*T*GGG, with a T substitution at A18]) that favored a major G-quadruplex structure (>95%) in K^+^ solution and gave an excellent NMR spectrum suitable for NMR structural determination (Fig. 1b and S2†).

The 1D ^1^H NMR spectra of the *htel21*T_18_ sample were recorded as a function of temperature. At 50 °C, the two peaks at ∼13.4 ppm and ∼9.6 ppm became broadened due to the exchange with the solvent, while the remaining 12 peaks remained sharp, suggesting that these two peaks did not belong to the G-tetrad core (Fig. S3a†).

The 1D ^1^H NMR spectrum of the *htel21*T_18_ sequence in K^+^ solution showed 12 well-resolved imino proton resonances at 10–12 ppm with sharp line widths (Fig. 1b), clearly indicating the formation of a predominant unimolecular G-quadruplex structure. Minor conformations were also present with peak intensities less than 5% when compared with those of the major species and thus did not interfere with the structural analysis of the predominant G-quadruplex structure.

To confirm the molecularity of *htel21* and *htel21*T_18_, we performed gel electrophoresis using dimeric 93del, d[GGGGTGGGAGGAGGGT], and the monomeric human telomere d[TAGGG(TTAGGG)_3_] as references. The bands corresponding to *htel21* and *htel21*T_18_ migrated at a similar position and faster than that of *h-telo*, indicating that both of these samples formed unimolecular G-quadruplex folds (Fig. S3b†).

### CD signature

The circular dichroism (CD) spectrum (Fig. 1c) of *htel21*T_18_ in K^+^ solution displayed two positive absorption peaks at ∼250 and ∼290 nm and a trough at ∼260 nm. The 290 nm peak was characteristic of opposite-polarity stacking of G-tetrads,^21^ suggesting that the sequence largely conforms to antiparallel G-quadruplexes in K^+^ solution.^20^,^22^,^23^ As shown in Fig. 1c, the CD spectrum of *htel21*T_18_ displayed a similar profile to that of *htel21*, except a shoulder for *htel21* at 270 nm probably caused by the conformational heterogeneity.

### Resonance assignment and glycosidic torsion angle determination of the *htel21*T_18_ G-quadruplex in K^+^ solution

The presence of 12 imino peaks in the 1D proton spectrum of *htel21*T_18_ in K^+^ solution (Fig. 1b) showed that all 12 guanines were involved in the intramolecular G-quadruplex formation and that this G-quadruplex structure contained three layers of G-tetrads. The imino and H8 protons of guanosine bases were unambiguously assigned through the low-enrichment (2%) ^15^N site-specific labelling method and 2D HMBC experiments (for more details about NMR assignments see Supplementary Results in the ESI†).

**Fig. 2:**
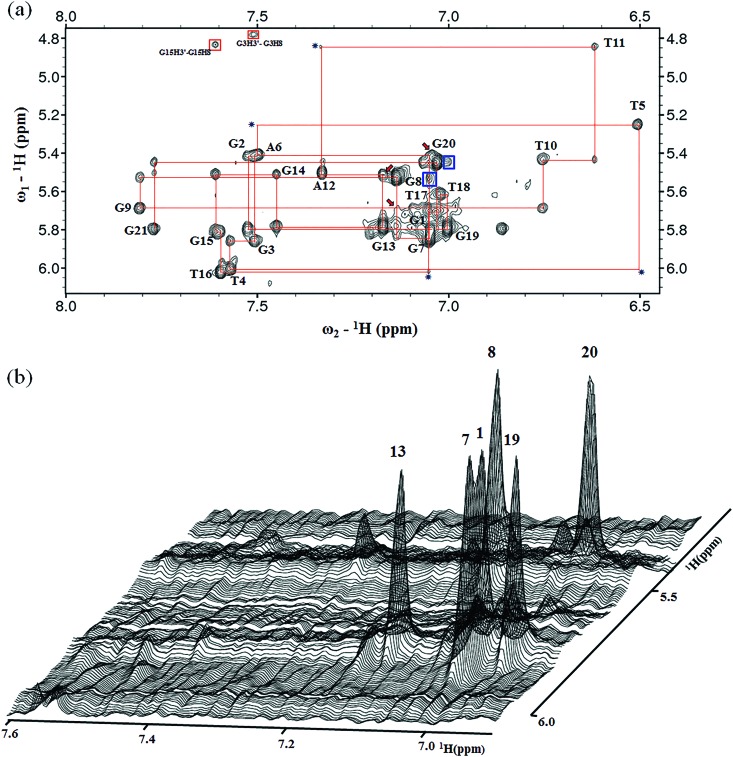
(a) NOESY spectrum (300 ms mixing time) showing the H6/H8–H1′ connectivity of *htel21*T_18_. The expanded ^1^H-^1^H NOESY spectrum (300 ms mixing time) correlates base H8 and sugar H1′ protons. Six guanine residues, including G1, G7, G8, G13, G19 and G20, are in the *syn*-conformation as indicated by very strong intraresidue guanosine H8–H1′ NOE connectivities. The *anti*G(*i*)H1′/*anti*G(i+1)H8 NOEs were observed, including G2–G3 and G14–G15. The *syn*G(i)H8/*syn*G(i+1)H1′ NOEs were observed, including G7–G8 and G19–G20 indicated with blue boxes. The characteristic *syn*G(i)H1′/*anti*G(i+1)H8 and *syn*G(i)H8/*anti*G(i+1)H1′ NOEs were observed, including G8–G9, G13–G14 and G20–G21. The characteristic NOE cross peaks for *syn*G(1)H8/*anti*G(2)H1′, *syn*G(8)H8/*anti*G(9)H1′ and *syn*G(13)H8/*anti*G(14)H1′ are indicated by red arrows. The missing or weak G(i)H1′/G(i+1)H8 cross peaks are indicated by a star. (b) Stacked plot of the short mixing time (75 ms) NOESY spectrum. The strong intraresidue guanosine H8–H1′ cross-peaks (*syn* glycosidic bonds) are labeled and can be distinguished from weak cross-peaks (*anti* glycosidic bonds).

An expanded region for base and sugar H1′ protons of the non-exchangeable proton NOESY spectrum is shown in Fig. 2a. Six strong cross-peaks in the H8–H1′ region of the 2D NOESY spectrum acquired at a 75 ms mixing time were interpreted as nucleotides with the *syn* conformation of glycosidic torsion angle, *i.e.*, G1, G7, G8, G13, G19 and G20 (Fig. 2b), in contrast to the other 6 guanines, namely, G2, G3, G9, G14, G15, and G21, that adopt the *anti* conformation in the quadruplex.

### Determination of the *htel21*T_18_ G-quadruplex folding topology

**Fig. 3:**
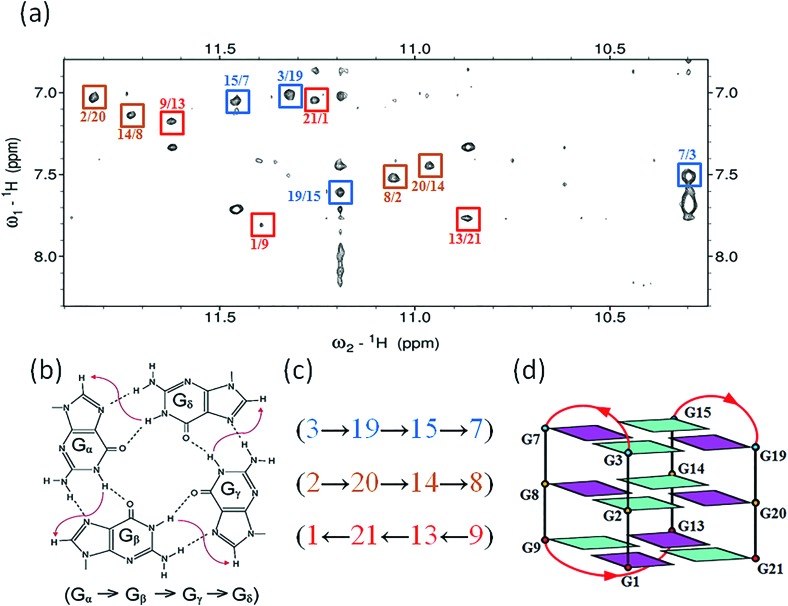
Determination of G-quadruplex topology for *htel21*T_18_ in K^+^ solution. (a) The NOESY spectrum (300 ms mixing time) of *htel21*T_18_ showing imino-H8 connectivity. Cross-peaks that identify the three G-tetrads are framed and labelled with the residue number of imino protons in the first position and that of H8 protons in the second position. (b) Characteristic guanine imino-H8 NOE connectivity patterns around a Gα·Gβ·Gγ·Gδ tetrad as indicated with arrows. (c) Guanine imino-H8 NOE connectivities observed for G1·G21·G13·G9, G2·G20·G14·G8 and G3·G19·G15·G7 tetrads. The bases in the G1·G21·G13·G9, G2·G20·G14·G8 and G3·G19·G15·G7 tetrads are colored red, orange, and blue, respectively. (d) Schematic structure of a chair-type G-quadruplex observed for a human telomeric variant DNA sequence, *htel21*T_18_, in K^+^ solution. *anti* guanines are colored cyan, while *syn* guanines are colored magenta. The backbones of the core and loops are colored black and red, respectively.

The assignment of the imino and base H8 protons of guanines in the 2D NOESY spectrum (mixing time 300 ms) (Fig. 3a) has led us to the direct determination of the folding topology of the G-quadruplex structure formed by *htel21*T_18_ in K^+^ solution. In a G-tetrad plane with a Hoogsteen-type H-bond network, the imino proton H1 of a guanine is in close spatial vicinity to the base H8 of one of the adjacent guanines (Fig. 3b). Analysis of characteristic NOEs between the imino and H8 protons revealed the formation of an intramolecular G-quadruplex involving three G-tetrads: G1·G21·G13·G9, G2·G20·G14·G8 and G3·G19·G15·G7. One external G-tetrad (G1·G21·G13·G9) is oriented with opposite hydrogen-bond directionality with respect to the other two G-tetrads (G2·G20·G14·G8 and G3·G19·G15·G7) (Fig. 3c). The hydrogen-bond directionalities of the three G-tetrads are anti-clockwise, clockwise and clockwise, respectively. The glycosidic conformations of guanines around the tetrads are *syn*·*anti*·*syn*·*anti*. The G-tetrad core is of antiparallel-type, in which each G-tract is oriented in the opposite direction with respect to its two neighboring ones. Connecting the corners of this G-tetrad core with linking sequences, we derived an antiparallel-stranded chair-type G-quadruplex fold (Fig. 3d). All three linkers in the structure, T4–T5–A6, T10–T11–A12 and T16–T17–T18, form edgewise loops.

### Assignment of the T4–T5–A6 and T16–T17–T18 loops of the *htel21*T_18_

As shown in Fig. 1, two extra peaks appeared with chemical shifts corresponding to ∼13.4 ppm and ∼9.6 ppm when A18 was substituted with T. Since all 12 guanines have been assigned through site-specific labelling, we hypothesized that these two peaks belonged to the T–T–A fragment. The chemical shift indicated that the peak at ∼13.4 ppm should be the imino proton involved in the H···N hydrogen bonding of the A·T pair.^24^ The peak at ∼9.6 ppm should be the imino proton involved in the H···O (donor–acceptor) type hydrogen bond or hydrogen bonding of the T–T pair.^25^,^26^ We chemically synthesized site-specific low-enrichment (2% ^15^N-labeled) DNA oligonucleotides for T4, T5, T10, T11, T16, T17 and T18 of *htel21*T_18_. The 1D ^15^N-filtered HSQC spectra showed that the peak at ∼13.4 ppm was the imino proton of T18, while T4, T10, T11 and T16 showed no signals (Fig. S4†). The peak at ∼9.6 ppm was the imino proton of T5 and the imino proton of T17 was close to G7 at ∼10.3 ppm (Fig. S4†). The characteristic NOEs observed in *htel21*T_18_ strongly supported the existence of the A6·T18 base pair (Fig. S5†).

### Overall solution structure of the *htel21*T_18_ G-quadruplex

**Fig. 4:**
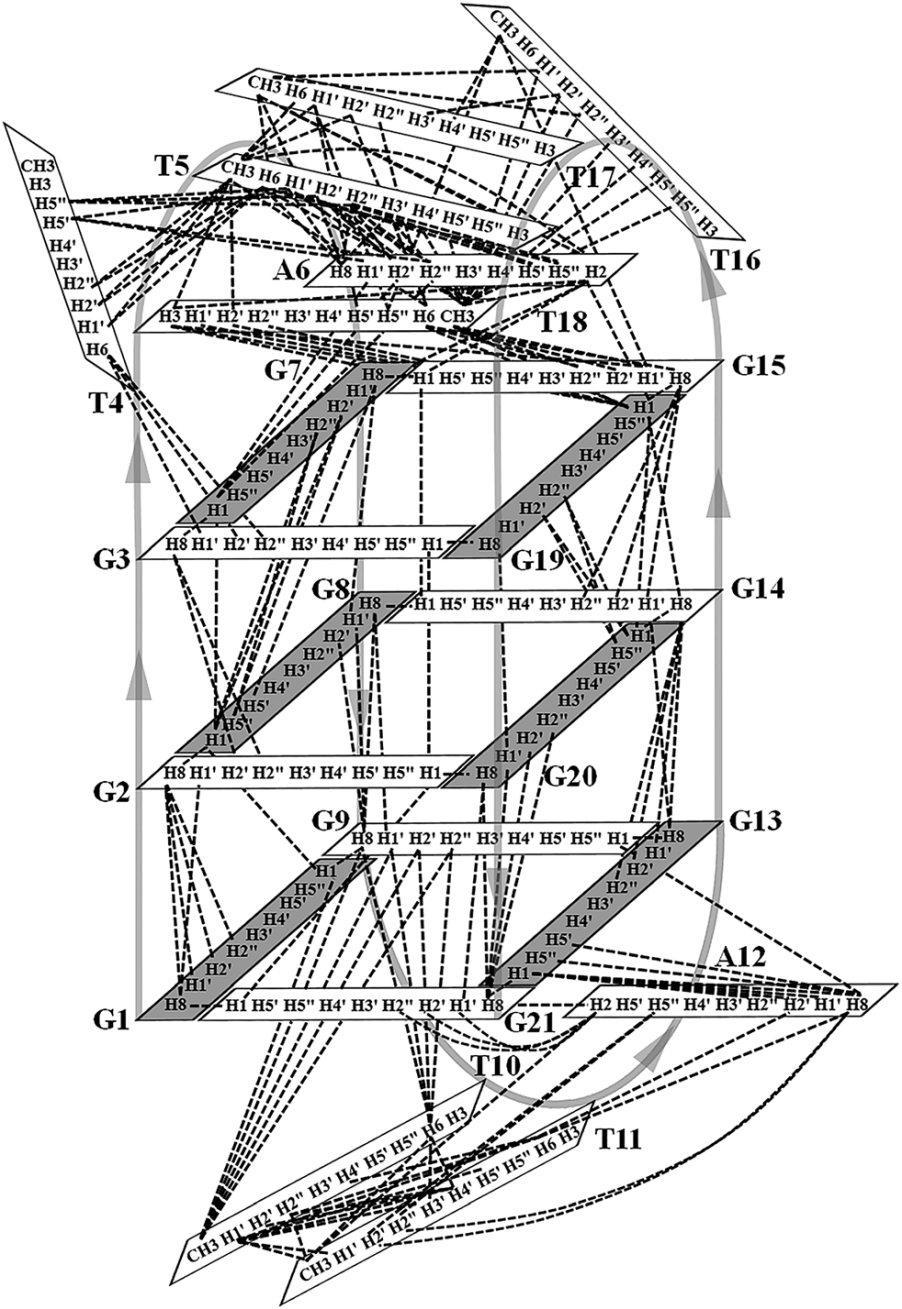
Schematic diagram of inter-residue NOE connectivities of the *htel21*T_18_ quadruplex formed in K^+^ solution. The guanines with the *syn* and *anti* conformations are represented using gray and white rectangles, respectively. The NOE connectivities clearly define the G-quadruplex conformation and provide distance restraints for structure calculation.

**Fig. 5:**
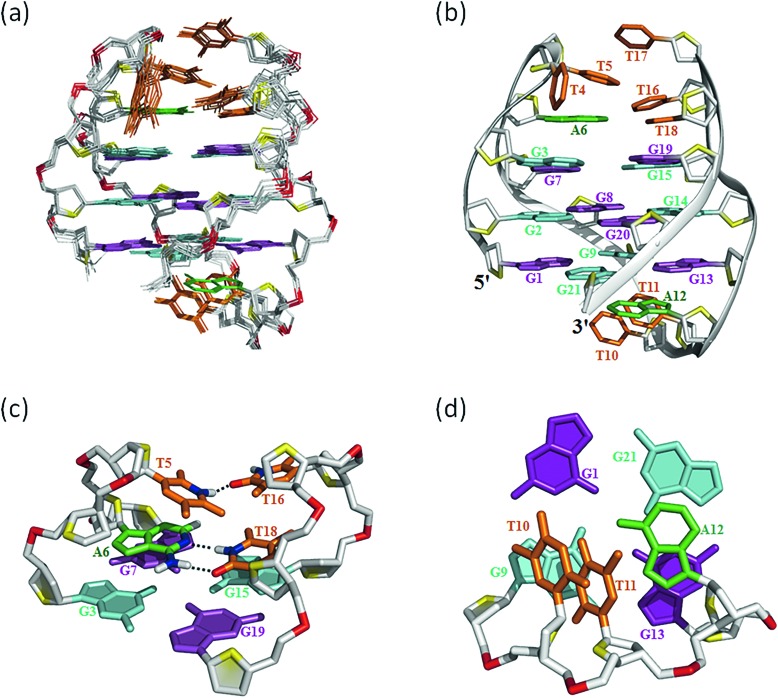
The *htel21*T_18_ G-quadruplex structure in K^+^ solution. (a) The 10 superimposed refined structures (PDB code: ; 5YEY). (b) Ribbon view of the representative lowest energy structure. *anti* and *syn* guanines are colored cyan and magenta, respectively; thymines are colored orange; adenines, green; backbone and sugar, gray; O4′ atoms, yellow; phosphorus atoms, red. Detailed loop structures of the *htel21*T_18_ G-quadruplex in K^+^ solution: (c) the conformation of loops T4–T5–A6 and T16–T17–T18. The hydrogen bond of the A6·T18 base pair and T5(H3)···T16(O4) is shown in dashed lines. (d) Representative loop conformation for the segment T10–T11–A12 of the lowest energy refined structure.

**Fig. 6:**
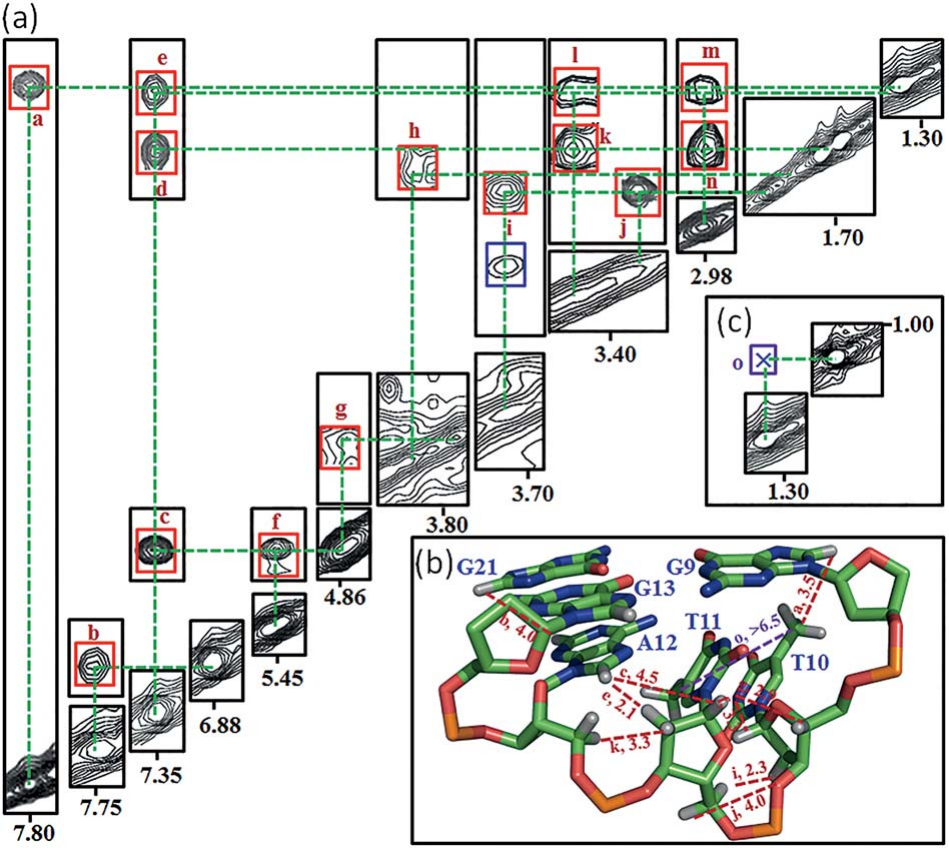
Analysis of the interaction between T10–T11–A12 and G1·G21·G13·G9 through NOEs observed in the NOESY spectrum of *htel21*T_18_ with a 300 ms mixing time recorded in D_2_O solution. (A) The observed and expected NOEs for the T10–T11–A12 loop with NOE assignments being listed in the contour plot. (B) Signature cross-peak plotted in the ribbon view of the representative lowest energy structure. (C) The cross peak between the methyl groups of T10 and T11. The corresponding NOE assignments: (a) G9H8-T10H7#, (b) G21H8-A12H2, (c) A12H8-T11H1′, (d) A12H8-T11H2′′, (e) A12H8-T11H2′, (f) T10H1′-T11H1′, (g) T11H1′-T10H4′, (h) T11H3′-A12H2′, (i) T115′-T10H2′′, (j) T115′′-T10H2′′, (k) A12H5′-T11H2′′, (l) A12H5′-T11H2′, (m) A12H5′′-T11H2′, (n) A12H5′′-T11H2′′, and (o) T10H7#-T11H7#. H7# is protons of the methyl group of the thymine.

Many inter-residue NOEs are observed in the 2D-NOESY spectrum of *htel21*T_18_ in K^+^ solution. Critical inter-residue NOEs are schematically summarized in Fig. 4. These NOEs define the overall structure of the telomeric G-quadruplex in K^+^ solution and were used for structure calculations. Ten superimposed lowest energy refined structures of the *htel21*T_18_ quadruplex are shown in Fig. 5a. The ribbon view of a representative refined structure of the *htel21*T_18_ quadruplex is shown in Fig. 5b. As shown in Fig. 5a, the G-quadruplex structure consists of three G-tetrads linked to form four antiparallel right-handed G-strands (G1–G2–G3, G7–G8–G9, G13–G14–G15 and G19–G20–G21) that are connected by three edgewise side loops (T4–T5–A6, T10–T11–A12, and T16–T17–T18). Both edgewise T4–T5–A6 and T16–T17–T18 loops are located on the same side of the G-quadruplex core. The edgewise T10–T11–A12 loop is on the opposite side of the G-quadruplex core. The structures of edgewise loops T4–T5–A6, T10–T11–A12 and T16–T17–T18 are well defined, partially because of the A6·T18 base pair and a hydrogen bond between the imino proton H3 of T5 and the oxygen atom O4 of T16 (Fig. 5c). Our structure suggests that T10–T11–A12 capping the G1·G21·G13·G9 tetrad may contribute to the stability of the structure (Fig. 5d). Experimentally, we observed numerous NOEs between the loop T10–T11–A12 and the G1·G21·G13·G9 layer, such as G9H8–T10H7# and G21H8–A12H2, as well as between the bases of the loop T10–T11–A12 such as A12H8–T11H1′, A12H8–T11H2′′, T10H1′–T11H1′ and others (Fig. 6a and b). The distance between the methyl groups of T10 and T11 is larger than 6.5 Å which corresponds to the absence of a cross peak between them in the NOESY spectrum (Fig. 6b and c). The experimental data are in full accordance with the conformation of the loop T10–T11–A12 in the structures.

### Substitution of A18 with T traps a chair-type *htel21*T_18_ G-quadruplex through the A6·T18 base pair

As shown in Fig. 5c and S6,† A6 and T18 form a reverse Watson–Crick *trans* A·T base pair which is almost parallel to the G-tetrad layer G3·G19·G15·G7. In the reverse Watson–Crick A·T base pair, the methyl group of T and the proton H2 of A should be on the same side. As shown in the schematic representation of inter-residue NOE contacts (Fig. 4), there are unambiguous NOEs such as CH3T18/H8G15, H2A6/H1G15 and H2A6/H8G7, indicating that the methyl group of T18 and the proton H2 of A6 are on the same side. These NOEs clearly demonstrate the formation of the reverse Watson–Crick A6·T18 base pair. Additionally, A6 and T18 are connected to G7 and G19 whose sugar-phosphate residues are parallel to each other. In this structural context, incorporation of a *cis* Watson–Crick pair is less feasible. Otherwise, it would have to be accompanied by a locally left-handed backbone configuration at the A6 → G7 step and a *syn* glycosidic torsion of the adenosine residue. However, none of these features are supported by experimental data.

This A6·T18 base pair formation results in a peak at ∼13.4 ppm originating from the imino proton of T18, a typical indicator of an imino proton hydrogen-bonded to a nitrogen acceptor (Fig. 1b). As shown in Fig. S5,† a NOE cross peak between H3 of T5 and the methyl group of T17 indicates that T5 and T17 are close in the G-quadruplex. In the calculated structure, there is a direct hydrogen bond between T5(H3) and T16(O4) in *htel21*T_18_ and T5 and T17 are almost capping the A6·T18 base pair and G3·G19·G15·G7 layer (Fig. 5c). The hydrogen bond T5(H3)···T16(O4) corresponds to a sharp imino proton signal of T5 at ∼9.6 ppm (Fig. S4†). The imino proton of T17 is responsible for the ∼10.3 ppm signal close to the imino proton of G7 (Fig. S4†). However, we did not observe any hydrogen bonds for the base T17 in our calculated structure. As shown in Fig. S6,† there may be a potential water-mediated hydrogen bond formed between the imino proton of T17 and a deoxyribose oxygen or phosphate group of T5. It is possible that a potential T·T base pair may exist even though the H3–H3 NOE cross peak between T5 and T17 could not be observed in the NOESY spectra with different mixing times. Additionally, the well-resolved 1D NMR spectra and similar melting temperatures, *T*_m_ of htel21T18 (∼73.1 °C) and htel21_A6T (∼74.0 °C) (Fig. S2 and S7†), indicate that the formation of the A·T base pair is important for this chair-type G-quadruplex fold. However, the slightly higher *T*_m_ of *htel21*_A6T could be possibly caused by the heterogeneity indicated by its 1D ^1^H spectrum in Fig. S2.†

Hence, the A·T base pair played an important role in trapping and stabilizing this chair-type G-quadruplex fold.

## Discussion

In this study, based on the bioinformatics search and with the use of CD and NMR spectroscopy, we have found a variant telomeric DNA *htel21*T_18_ that has a T substitution at A18 of *htel21* and showed that it adopts a chair-type G-quadruplex fold with three G-tetrad layers. As shown in Fig. 1b and S2,† htel21T18 favoured a major G-quadruplex structure and gave excellent NMR spectra suitable for NMR structural determination. Both the 1D 1H NMR spectrum and CD spectrum clearly indicated the formation of a predominant three G-tetrad layered, antiparallel G-quadruplex structure by the *htel21*T_18_ (Fig. 1). Stabilization of its loop–loop interactions by a reverse Watson–Crick A6·T18 base pair allows one to predict that an A6T mutant could form a similar structure, with the T6·A18 base pair with the same geometry in its two opposite lateral loops. Indeed, the 1D ^1^H spectra of *htel21*_A18T and *htel21*_A6T are very similar (Fig. S2b†). Besides *htel21*T_18_, two *htel21* variants obtained by substituting T4 and T10 with A also favoured a single G-quadruplex form. However, the number of imino protons observed between 10.0 and 12.5 ppm indicated that the G-quadruplexes adopted by *htel21*_T4A and *htel21*_T10A contain two, not three, G-tetrad layers. Comparison with the ^1^H spectra of reported human telomeric G-quadruplexes in ESI Fig. S1† allows one to expect that the topology of *htel21*_T4A and *htel21*_T10A may be similar to that of the intramolecular basket-type G-quadruplex formed by the sequence d[(GGGTTA)_3_GGGT].^17^

It is known that base pairing and stacking in the loops often serve as stabilizing factors or affect the selection of a particular one among several possible forms for G-quadruplexes.^27^ In the current structure of the chair type G-quadruplex of *htel21*T_18_, the A6·T18 base pair is observed across two juxtaposed lateral loops capping the G-tetrad core on one side. We note, however, that the G-core in the reported quadruplex topology is stable and sustains heating up to 50 °C, the temperature at which the A6· T18 base pair is already melted (Fig. S3a†). In the structure *htel21*T_18_, there is also a hydrogen bond T5(H3)···T16(O4), and T5 and T17 are almost capping the A6·T18 base pair and underlying G3·G7·G15·G19 layer (Fig. 5c).

Since the imino proton of T17 shows a peak at ∼10.3 ppm, but no suitable hydrogen bond is observed in our calculated structure, it might be an indication of a water-mediated hydrogen bond with an opposing oxygen on sugar-phosphate (Fig. S6†). We hypothesize that potential hydrogen bonds of the T·T base pair involving T17 are helpful in stabilizing the chair-type G-quadruplex fold. As far as the T10–T11–A12 loop is concerned, this fragment converged well in the ensemble of computed structures (Fig. 6), suggesting that the particular geometry adopted by single edgewise loop T10–T11–A12 is energetically favorable and its stacking interactions with neighbouring G1·G21·G13·G9 may contribute to the stability of the structure as well (Fig. 5d). The conformations of nucleotides T4 and T16 are predominantly defined by van der Waals interactions which position them in a rather restricted volume defined by the structured covalently bound neighbouring nucleotides (Fig. 5a and S8†).

Subtelomeric occurrence in the human genome of the sequence *htel21*T_18_ (Table S1†), which is strongly prone to the chair-type quadruplex described here, indicates that this variant of human telomeric DNA may result from a single residue mutation and as such may change the equilibrium of G-quadruplex forms on pearls-on-string single-stranded G-telomeric overhangs, with accompanying changes in topologies and relative orientations of monomeric quadruplex units on such a string. The sequence *htel21*T_18_ does not exclusively localize in sub-telomeric regions, but is also found in other regions, such as in the subcentromeric region of chromosome 5. Interestingly, the occurrence of this sequence in a DNase hypersensitive region implies that in this part of chromosome a chair-like quadruplex can easily form *in vivo*.

Until now, several chair-type G-quadruplex structures have been reported such as thrombin aptamer d(G_2_T_2_G_2_TGTG_2_T_2_G_2_), a sequence variant of the human telomeric sequence, d[AGGG(CTAGGG)_3_], and a *Bombyx mori* telomeric sequence, *Bm-U16*, which are all composed of two antiparallel G-tetrad layers.^28^–^31^ In comparison with *htel21*T_18_, these structures are different in the loop length, the hydrogen-bond directionalities of the G-tetrad layers, *etc.* Recently, a four-layer antiparallel G-quadruplex in which the symmetry and strand orientation are similar to *htel21*T_18_ has been reported to be formed by an intronic hexanucleotide GGGGCC (G4C2) repeat of the C9orf72 gene in humans^32^,^33^ (for more details see ESI†).

## Conclusions

In summary, we have determined a novel three-layer chair type G-quadruplex structure of a human telomeric variant DNA *htel21*T_18_. The unique structure and fold of *htel21*T_18_ could enable selective recognition and binding of a ligand and may provide a potential target for the development of a specific drug molecule stabilizing this DNA conformation. Our result expands the repertoire of known G-quadruplex folding topologies and highlights the important role of the loops on the folding topology of G-quadruplexes.

## Experimental

### Sample preparation

DNA synthesis was performed on a 1 μmol scale, using a 1000 Å LCAA-CPG solid support column on a MerMade 6 Nucleic Acid Synthesiser. Unlabeled and site-specific low-enrichment (2% ^15^N- or 7% ^15^N, ^13^C-labeled) nucleotides were site-specifically introduced into the growing oligonucleotide chains. All sequences were fully deprotected in concentrated ammonium hydroxide at room temperature for 24 hours. The DNA samples were dried, redissolved in ∼1 mL water and purified by gel-filtration chromatography on a Sephadex G-25 column. The DNA sample at 100 μM (single strands) was then re-annealed by heating to 95 °C for 15 min, followed by slow cooling to room temperature overnight in an annealing buffer of 70 mM KCl and 20 mM potassium phosphate (pH 7.0). The final NMR samples contained 0.1–2.5 mM DNA in 20 mM potassium phosphate buffer (pH 7.0) and 70 mM KCl.

### Circular dichroism

Circular dichroism (CD) spectra were recorded at 25 °C on a JASCO-815 CD spectropolarimeter using a 1 mm path length quartz cuvette with a sample volume of 200 μl. The DNA oligonucleotides were prepared in 20 mM potassium phosphate buffer (pH 7.0) containing 70 mM KCl at a concentration of 20 μM (strands).

### Polyacrylamide gel electrophoresis (PAGE)

Non-denaturing PAGE was carried out in 25% polyacrylamide gel (acrylamide : bis-acrylamide 29 : 1), supplemented with 20 mM KCl in the gel and running buffer (TBE 0.5×). The samples were prepared at a strand concentration 100 μM. Bands were stained with Red-safe dye.

### NMR spectroscopy

Experiments were performed on 500 MHz and 800 MHz Varian spectrometers. Imino proton resonances were assigned to samples with nucleotides site-specifically ^15^N labeled, one at a time, and by through-bond correlations at natural abundance.^34^,^35^ Standard 2D NMR experimental spectra, including NOESY, TOCSY and COSY, were collected at 5, 10 and 25 °C to obtain the complete proton resonance assignment.^36^ The NMR experiments for samples in water solution were performed with Watergate or Jump-and-Return water suppression techniques. Spectra were processed with the program nmrPipe.^37^,^38^ Spectral assignments were also carried out and supported by COSY, TOCSY and NOESY spectra. NOE peak assignments and integrations were made using peak fitting and volume integration implemented in the software Sparky (; http://www.cgl.ucsf.edu/home/sparky/). Interproton distances involving exchangeable protons for *htel21*T_18_ were categorized as strong (1.8 to 3.8 Å), medium (1.8 to 4.5 Å), weak (2.8 to 5.5 Å) or very weak (2.8 to 6.8 Å) based on the cross-peak intensities recorded in three NOESY spectra (75, 150 and 300 ms mixing times) in H_2_O solution. Interproton distances involving non-exchangeable protons for *htel21*T_18_ were measured from NOE build-ups using NOESY experiments recorded at three mixing times (75, 125, and 300 ms) in D_2_O solution. The thymine base proton H6–H7# distance (2.99 Å) was used as a reference distance.

### Structure calculations

The G-quadruplex structure of the sequence d[(GGGTTA)_2_GGGTT*T*GGG] was calculated using the X-PLOR program (NIH VERSION).^39^,^40^ The initial folds guided by NMR restraints listed in Table S2† were obtained using torsion angle dynamics from an arbitrary extended oligonucleotide conformation. The structures were further refined by Cartesian dynamics.^41^ Dihedral angle restraints were used to restrict the glycosidic torsion angle (*χ*) for the experimentally assigned *syn*- and *anti*-conformations, 60(±35)° and 240(±40)°, respectively.^42^–^44^ Experimentally obtained distance restraints and G-tetrad hydrogen-bonding distance restraints were included during calculations.

### Torsion angle dynamics

In the heating stage, the regularized extended DNA chain was subjected to 60 ps of torsion-angle molecular dynamics at 40 000 K using a hybrid energy function composed of geometric and NOE terms. The van der Waals (vdW) component of the geometric term was set at 0.1, while the NOE term included NOE-derived distances with a scaling factor of 150. The structures were then slowly cooled from 40 000 K to 1000 K over a period of 60 ps during which the vdW term was linearly increased from 0.1 to 1. In the third stage, the molecules were slowly cooled from 1000 K to 300 K for 6 ps of Cartesian molecular dynamics.^45^ The structures with no restraint violations and minimal energies were selected for further refinement.

### Distance restrained molecular dynamics

Cartesian molecular dynamics was initiated at 300 K and the temperature was gradually increased to 1000 K in 6 ps. The system was equilibrated at 1000 K for 18 ps and was then slowly cooled to 300 K in 14 ps. Subsequently, the system was equilibrated at 300 K for 12 ps. The coordinates saved every 0.5 ps during the last 4.0 ps were averaged. The resulting average structure was subjected to minimization until the gradient of energy was less than 0.1 Kcal mol^–1^. A soft planarity restraint (weight of 10 kcal mol^–1^ Å^–2^) was imposed on the G-tetrads before the heating process and was removed at the beginning of the equilibration stage. 10 best structures were selected at this stage based on both their minimal energy terms and without NOE violations. The statistics of the structure refinement and the quality of the final structures are summarized in Table S2† for *htel21*T_18_. The proton chemical shifts of *htel21*T_18_ are shown in Table S3.†

All images of G-quadruplex structures in the figures were generated using PyMOL (http://www.pymol.org).

### Genome screening for sequence occurrence

We screened the human genome using a single sequence repeat as the input for BLAT search (http://genome.ucsc.edu/).

### Data deposition

The atomic coordinate has been deposited at the Protein Data Bank for the sequence d[(GGGTTA)_2_GGGTT*T*GGG] (accession code ; 5YEY).

## Conflicts of interest

There are no conflicts to declare.

## Supplementary Material

Supplementary information
