# Supplementary material for: A chair-type G-quadruplex structure formed by a human telomeric variant DNA in K+ solution
Source: Chem Sci. 2018 Oct 4;10(1):218–26. doi: 10.1039/c8sc03813a (PMC6330691; doi:10.1039/c8sc03813a)
Supplement: Supplementary file 1 [file SC-010-C8SC03813A-s001.pdf]

Electronic Supplementary Material (ESI) for Chemical Science.
This journal is © The Royal Society of Chemistry 2018

Supporting information

A Chair-type G-quadruplex Structure Formed by a Human Telomeric Vatiant DNA Sequence in K+ solution

Changdong Liu[1*], Bo Zhou[1,2*], Yanyan Geng[1*], Dick Yan TAM[3], Rui Feng[1], Haitao Miao[1], Naining Xu[1], Xiao Shi[1], Yingying You[1], Yuning Hong[4], Benzhong Tang[4], Pik Kwan LO[3], ,Vitaly Kuryavyi[5‡] and Guang Zhu[1,6‡]

[1]Division of Life Science, The Hong Kong University of Science and Technology, Clear Water Bay, Kowloon, Hong Kong SAR, China.

[2]Institute for Advanced Study, The Hong Kong University of Science and Technology, Clear Water Bay, Kowloon, Hong Kong SAR, China.

[3]Department of Biology and Chemistry, City University of Hong Kong, 83 Tat Chee Avenue, Kowloon Tong, Hong Kong SAR, China.

[4]Department of Chemistry, The Hong Kong University of Science and Technology, Clear Water Bay, Kowloon, Hong Kong SAR, China.

[5]Structural Biology Program, Memorial Sloan-Kettering Cancer Center, New York, NY, USA.

[6]Institute for Advanced Study and State Key Laboratory of Molecular Neuroscience, The Hong Kong University of Science and Technology, Clear Water Bay, Kowloon, Hong Kong SAR, China.

*: These authors contributed equally to the work.

‡Corresponding author: v.kuryavyi@gmail.com; gzhu@ust.hk

SUPPLEMENTARY RESULTS

Resonance assignments and glycosidic torsion angle determination of the *htel21*T_{18} G-quadruplex in K+ solution

In order to unambiguously assign imino proton resonances, low-enrichment (2%) ^{15}N site-specific labelled oligonucleotides were synthesized and 1D ^{15}N-filtered HSQC spectra were recorded. One signal was observed in the 1D ^{15}N-filtered HSQC spectrum for the imino proton of the isotopically labelled guanine base in each sample (Fig. S9a†). Identified imino signals of 1D 1H-NMR spectra allowed further assignment of proton resonances by 2D NMR methods.

A 2D HMBC experiment was performed to correlate guanine H8 and H1 protons within the same guanine base and thus assigning H8 protons of guanine bases (Fig. S9b†). For assignments of thymine bases, low-enrichment (7%) 15N, 13C site-specific labelled oligonucleotides were synthesized and 2D ^{13}C-HSQC spectra were recorded (Figs. S10a and S10b†). The H2'/H2'' assignments were performed via strong intra-NOE H2'-H2'' and, in addition, by stronger H1'/H2'' versus H1'/H2' cross peaks of nucleotides.

As shown in Fig. 2, G1, G7, G8, G13, G19 and G20 adopt with the *syn* conformation of glycosidic torsion angle. While other 6 guanines, G2, G3, G9, G14, G15, G21, adopt *anti* conformation in quadruplex. *Anti-anti* and *syn-syn* steps of guanine nucleotides in a quadruplex have different signatures, and are distinguished by G(i)H1'/G(i+1)H8 and G(i)H8/G(i+1)H1' NOEs, respectively. For *syn-anti* connection, both the *syn*G(i)H1'/*anti*G(i+1)H8 and *syn*G(i)H8/*anti*G(i+1)H1' NOEs are observable.[1] In current study, as shown in Fig. 2a, the regular sequential NOE connectivities for *anti*G(i)H1'/*anti*G(i+1)H8 NOEs were observed, including *anti*G2-*anti*G3 and *anti*G14-*anti*G15. The characteristic *syn*G(i)H8/*syn*G(i+1)H1' NOEs were observed, too, including *syn*G7-*syn*G8 and *syn*G19-*syn*G20. The sequential *syn*G(i)H1'/*anti*G(i+1)H8 and *syn*G(i)H8/*anti*G(i+1)H1' NOEs were observed as well, including *syn*G8-*anti*G9, *syn*G13-*anti*G14 and *syn*G20-*anti*G21. H8/H1' of *syn*G1-*anti*G2 and *syn*G20-*anti*G21 were not so well resolved due to the chemical shift overlaps, also with H8 and H1'-proton frequencies of G7 and G19 (Fig. 2a).

Although the chemical shifts of H8 protons of G1, G7 and G19 are very close, three separated intraresidue cross peaks H8-H1' of G1, G7 and G19 could be clearly identified in a NOESY spectrum with 75 ms mixing time (Fig. S11†). Especially, characteristic *syn*G(i)H8/*syn*G(i+1)H1' NOEs were observed for *syn*G7-*syn*G8 and *syn*G19-*syn*G20 steps. The intra H8-H1' cross peaks of G1, G7 and G19 were thus unambiguously assigned. All the chemical shifts of H6/H8 protons were verified through aromatic ^{13}C-HSQC spectra (Fig. S10c). The sequential walk was confirmed by the H2'/H2''-H6/H8 NOE data.

For the H2 protons of A6 and A12, apparently two isolated peaks can be clearly assigned to H2C2 of A6 and A12 in the 13C-HSQC spectra locating between ~152ppm to ~159ppm, (Fig. S10d†). The H2C2 of A6 and A12 can be unambiguously identified through the NOE cross peaks such H2A6-H8G7 and H2A12-H8G21.

Other sugar proton resonances were identified and assigned with the use of 2D COSY, TOCSY and NOESY spectra. The resonances of thymine methyl groups were identified through a 2D COSY spectrum. Based on the intra-base cross peak (H6-H1' of thymines) in 2D NOESY spectra and in combination with unambiguously assigned H8, H1' of guanines, the methyl groups H7#, H6 and H1' of thymines were assigned through already established at a previous stage of data analysis NOE-based sequential walk (Fig. 2a). After H1' of thymines were identified, other protons of sugars including H2', H2'', H3', H4', H5', H5'' were assigned based on COSY, TOCSY and NOESY spectra. Due to the severely overlapped cross peaks and very low peak intensities between H2' and H3', H4', H5', H5'' of the guanine bases in COSY and TOCSY spectra, some of the assignments of H3', H4', H5', H5'' of guanine bases cannot be assigned unambiguously.

SUPPLEMENTARY DISCUSSION

The chair type G-quadruplex structure of *htel21*T_{18} involves three-layer antiparallel G-tetrad core and three edgewise loops (Fig. 5). Base pairing and stacking of nucleotides in these loops, T4-T5-A6 and T16-T17-T18, contribute to stabilization of this G-quadruplex form (Fig. 5). As shown in the Supplementary Fig. S1†, two antiparallel-stranded basket-type forms are known for human telomeric sequence. One is formed by d[A(GGGTTA)$_3$GGG] sequence with three G-tetrad layers in Na+ solution (Fig. S1a†), and the other , with d[(GGGTTA)$_3$GGGT] sequence, has two G-tetrad layers in K+ solution (Fig. S1e†). Two lateral loops in these structures run in parallel orientation with respect to each other, while in the structure reported here these loops have antiparallel orientation. Both basket-type G-quadruplex structures contain three loops, which are successively edgewise, diagonal, edgewise. In the structure of d[A(GGGTTA)$_3$GGG], T6 and A19 are almost parallel to and stack over the G-tetrad plane in a position that allows them to form hydrogen bonds (Fig. S1a†). In the structure of *htel21*T_{18}, the position of A6•T18 base pair is simalir with the T6 and A19 in the structure of d[A(GGGTTA)$_3$GGG], except the loop polarity aspect noticed already. In the structure of d[(GGGTTA)$_3$GGGT] sequence, the bases in the two edgewise loops interact through a base triple A•G•A and possible T•T base pair which stack over the G-tetrad plane. These data further indicate the importance of loop-loop interactions and, particularly, the effect of A6•T18 base pair on the chair-type G-quadruplex in the human telomeric sequence.

Previously, a chair-type G-quadruplex structure has been reported for thrombin aptamer d($G_2T_2G_2TGTG_2T_2G_2$), which forms a two-layered quadruplex with shorter loops composed exclusively of T residues[2, 3]. More recently, another chair-type quadruplex structure adopted by a sequence variant of human telomeric sequence, d[AGGG(CTAGGG)$_3$], with all TTA loops replaced by CTA (termed 22CTA) has been reported, which is composed of two antiparallel G-tetrad layers and a G•C•G•C tetrad connected by three edgewise loops [4]. In our structure presented here, all tetrads are formed by G nucleotides, and, in a sequence variant, only one residue out of three edgewise loops bears a non-telomeric A to T substitution. The hydrogen-bond directionalities of the three G-tetrad layers are anti-clockwise, clockwise and clockwise, and not anti-clockwise, clockwise and anti-clockwise (or clockwise, anti-clockwise and clockwise) as in two other proposed models[5, 6]. The difference between *htel21*T_{18} and 22CTA is in the composition of the third layer and in the loop length. The two guanines in the third layer (G•C•G•C tetrad) of 22CTA are all in *anti* confrmations. The corresponding two guanines in G3•G19•G15•G7 layer of *htel21*T_{18} are in *syn* conformations. For the loop length, there are two short TA loops and one CTAG loop in 22CTA. In *htel21*T_{18}, however, the three loops are of the same 3-residue length. Another chair type quadruplex structure formed by a *Bombyx mori* telomeric sequence, *Bm-U16*, was reported [7]. The G-quadruplex structure of *Bm-U16* contains a two-layer antipaprallel G-tetrad core and three egdewise loops. Symmetry and directionality of two G-tetrads in *Bm-U16* resembles these in two out of three layers in *htel21*T_{18}.

SUPPLEMENTARY FIGURES

Figure S1 Schematic structures of intramolecular G-quadruplexes formed by four-repeat human telomeric sequences: (a) The basket-type form observed for d[A(GGGTTA)$_3$GGG] in Na+ solution, (b) the propeller-type form observed for d[A(GGGTTA)$_3$GGG] in a K+ containing crystal, (c) the (3 + 1) Form 1 observed for d[TA(GGGTTA)$_3$GGG] in K+ solution, (d) the (3+1) Form 2 observed for d[TA(GGGTTA)$_3$GGGTT] in K+ solution, (e) the basket-type form with two G-tetrad layers observed for the modified d[(GGGTTA)$_3$GGGT] sequence in K+ solution, and (f) the antiparallel (2+2) form observed for d[(TTAGGGTTA)$_4$TTA] G-quadruplex in Na+ solution, (g) the (3 + 1) Form 1 observed for the end-modified sequence d[AAA(GGGTTA)$_3$GGGAA] in K+ solution, (h) the (3+1) Form 2 observed for d[TTA(GGGTTA)$_3$GGGTT] sequence in K+ solution. *anti* guanines are colored in cyan, while *syn* guanines are colored in magenta.

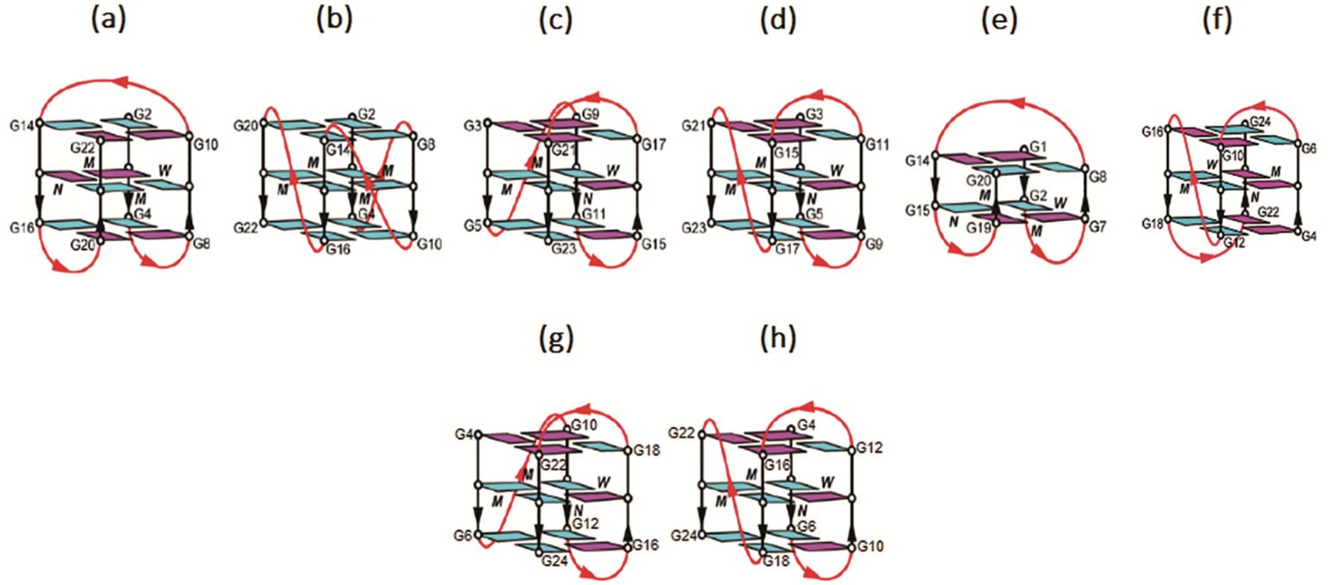

Figure S2 (a) Native and variant four-G-tract human telomeric DNA sequences found by BLAST search. (http://genome.ucsc.edu/). (b) imino regions of 1D ^{1}H NMR spectra for the DNA sequences shown in (a).

(a).

Name	***Sequence***			
htel21	1_GGGTTA	GGGTTA	GGGTTA	GGG_21
*htel21*_T4A	1_GGGA$_4$TA	GGGTTA	GGGTTA	GGG_21
*htel21*_T5A	1_GGGTA$_5$A	GGGTTA	GGGTTA	GGG_21
*htel21*_T4A-T5A	1_GGGA$_4$A$_5$A	GGGTTA	GGGTTA	GGG_21
*htel21*_A6T	1_GGGTTT$_6$	GGGTTA	GGGTTA	GGG_21
*htel21*_T10A	1_GGGTTA	GGGA$_{10}$TA	GGGTTA	GGG_21
*htel21*_T11A	1_GGGTTA	GGGTA$_{11}$A	GGGTTA	GGG_21
*htel21*_A12T	1_GGGTTA	GGGTTT$_{12}$	GGGTTA	GGG_21
*htel21*_T16A	1_GGGTTA	GGGTTA	GGGA$_{16}$TA	GGG_21
*htel21*_T17A	1_GGGTTA	GGGTTA	GGGTA$_{17}$A	GGG_21
*htel21*_A18T	1_GGGTTA	GGGTTA	GGGTTT$_{18}$	GGG_21

(b).

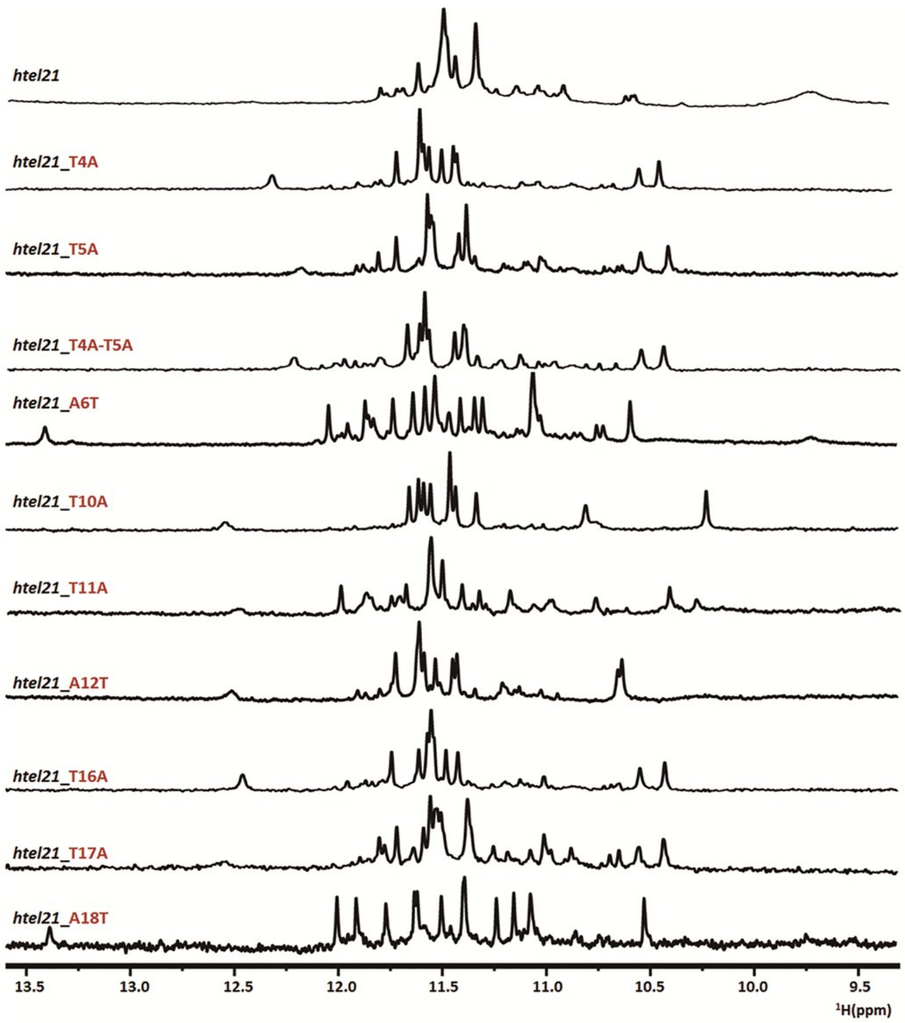

Figure S3 (a**)** Imino proton spectra of *htel21*T_{18} at 10 ºC, 30 ºC, 50 ºC. The peaks for T5 and T18 are labeled in red color. (**b)** Electrophoretic mobilities of *htel21* and *htel21*T_{18} sequences are compared with dimeric 93del, d[GGGGTGGGAGGAGGGT],[8] and monomeric human telomere, d[TAGGG(TTAGGG)$_3$], named as *h-telo*.[9] All the samples were run in non-denaturing 25% PAGE at 100 µM concentration.

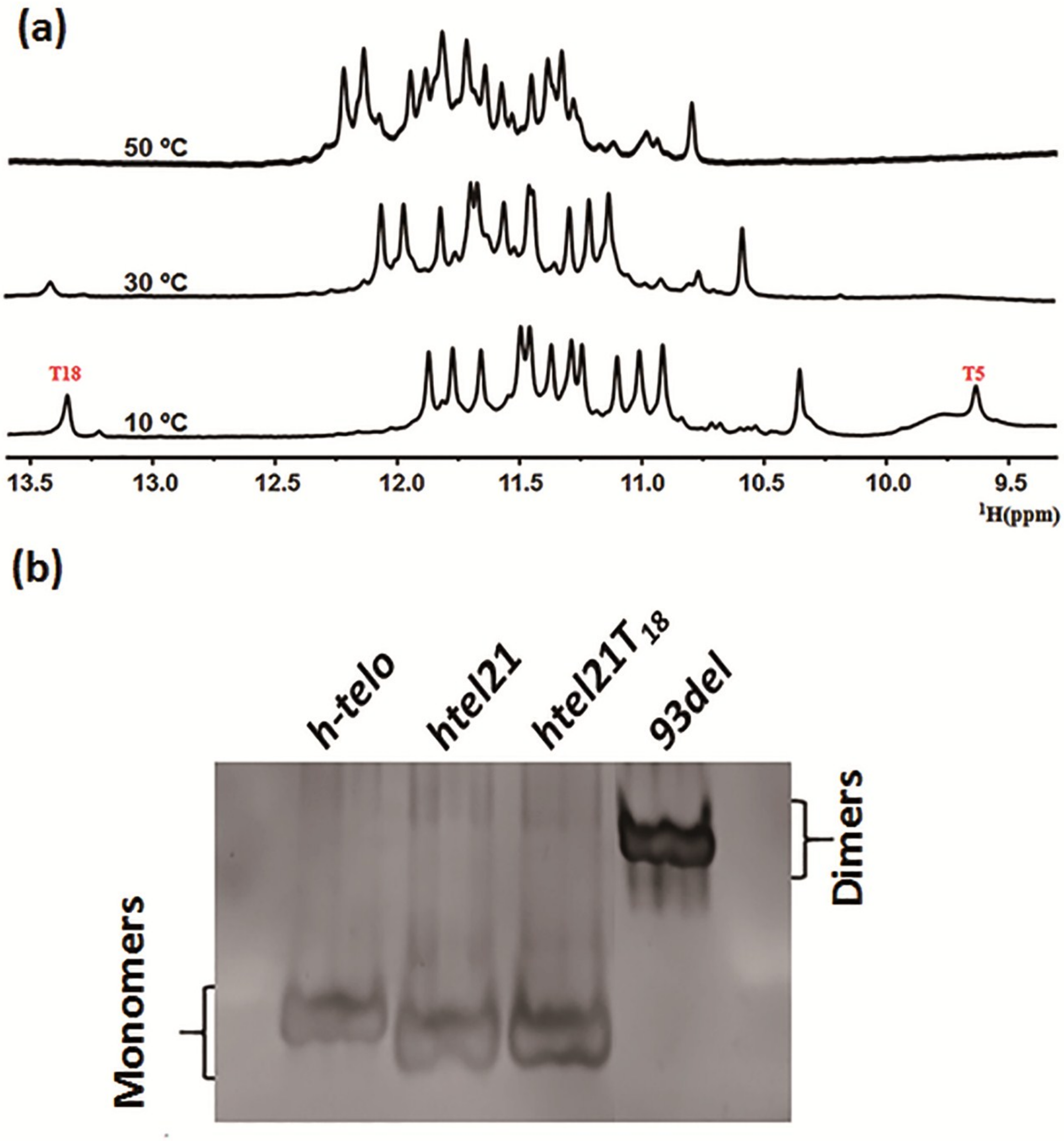

Figure S4 The imino regions of 1D ^{15}N-filtered HSQC spectra of *htel21*T_{18} samples containing site-specific low-enrichment (2%) ^{15}N labelled oligonucleotides at indicated positions 4, 5, 10, 11, 16, 17 and 18. Spectra were recorded at 800 MHz (1D ^{1}H-NMR spectrum and 1D ^{15}N filtered HSQC spectra) at 10 °C in 5% D_2O, 70 mM KCl, 20 mM potassium phosphate buffer with pH 7.0. The 1D ^{1}H-NMR reference spectrum is on the top.

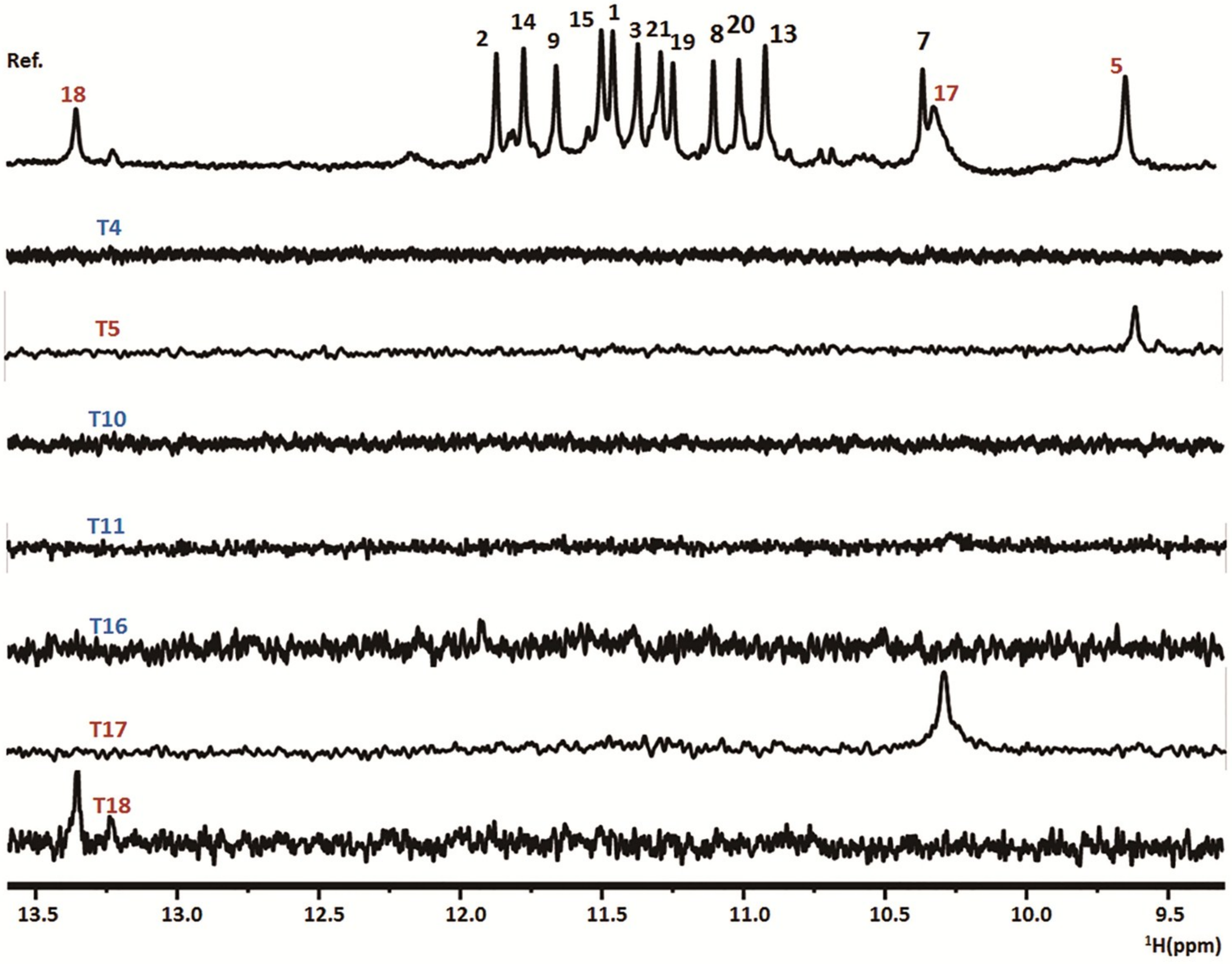

Figure S5 T5 and T18 strip plots of the 2D ^{1}H NOESY spectrum (300 ms mixing time) of *htel21*T_{18} in water. Green and red labels correspond to the A•T base pair specific correlations.

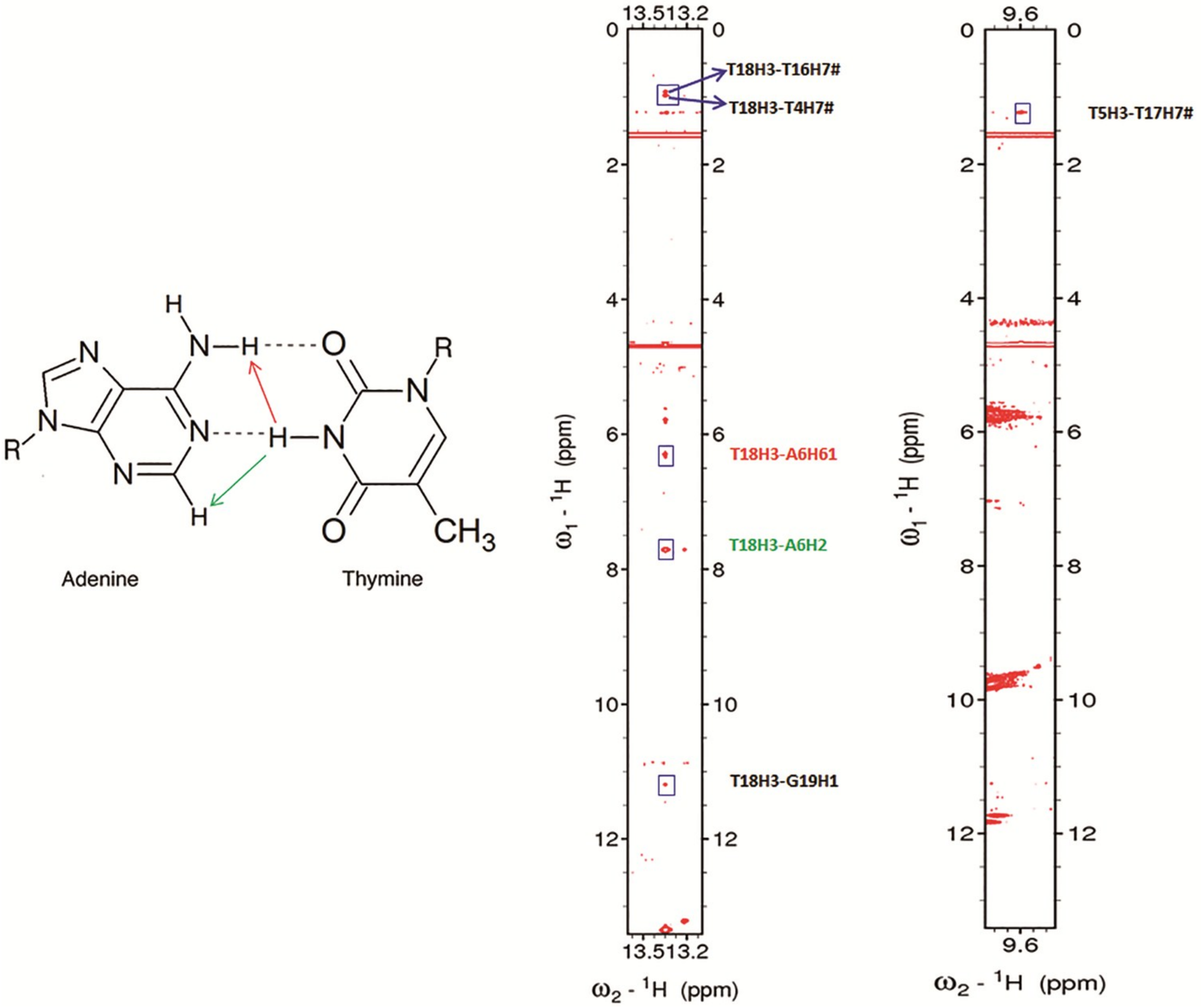

Figure S6 The detailed structure of T5, T17 and the A6•T18 base pair of *htel21*T_{18}. The distance between H3 of T5 and the oxygen atom O4 of T16, T5(H3)…T16(O4), is labelled. The hydrogen bonds are shown in dashed line.

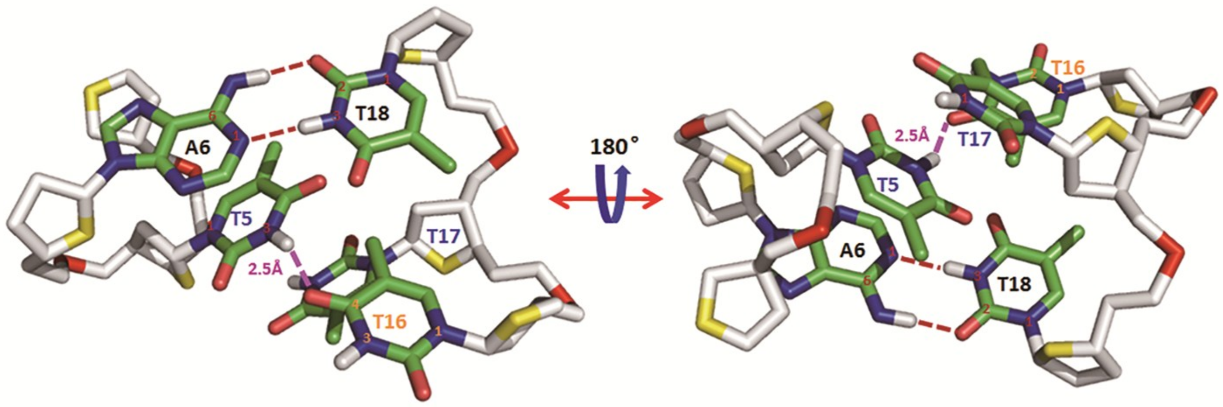

Figure S7 The CD melting experiments of *htel21*, *htel21*_A6T and *htel21*_A18T (named as *htel21*T_{18} in this study). The sequences and the melting temperatures, T_ms, are listed below.

Name	***Sequence***				T_m **(℃)**
htel21	1_GGGTTA	GGGTTA	GGGTTA	GGG_21	73.5
*htel21*_A6T	1_GGGTTT_6	GGGTTA	GGGTTA	GGG_21	74.0
*htel21*_A18T	1_GGGTTA	GGGTTA	GGGTTT_{18}	GGG_21	73.1

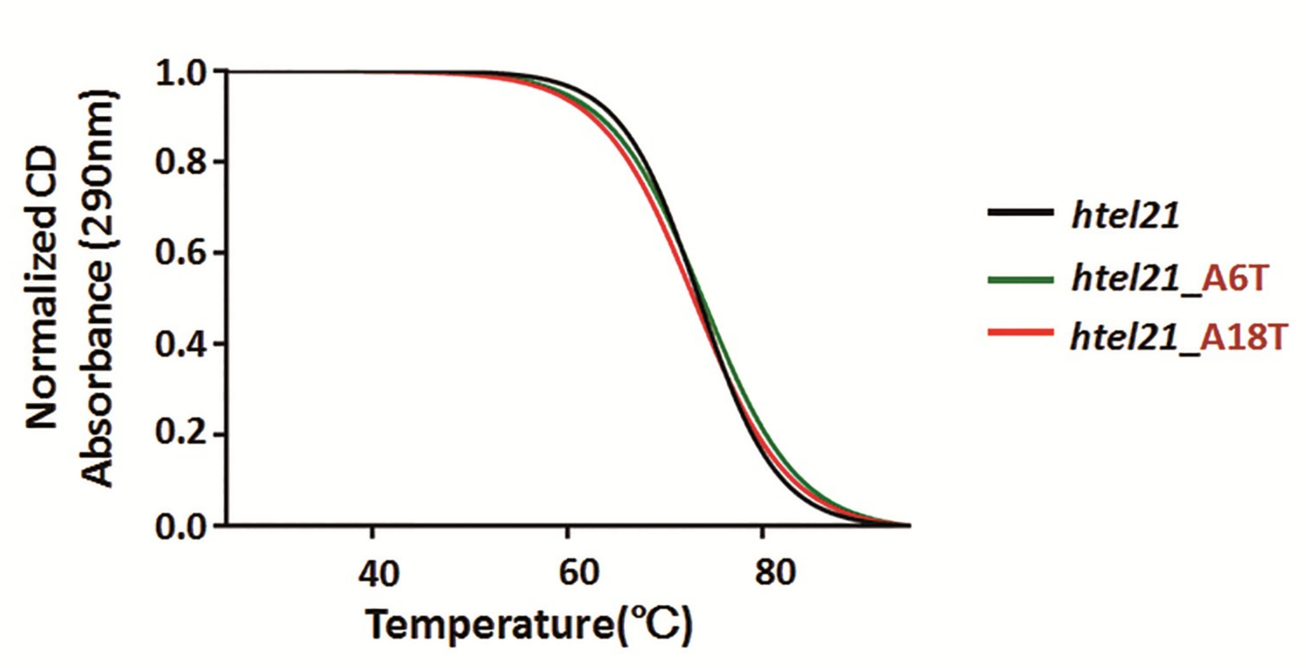

Figure S8 The conformation of T4, T16 and G3•G19•G15•G7 in *htel21*T_{18}

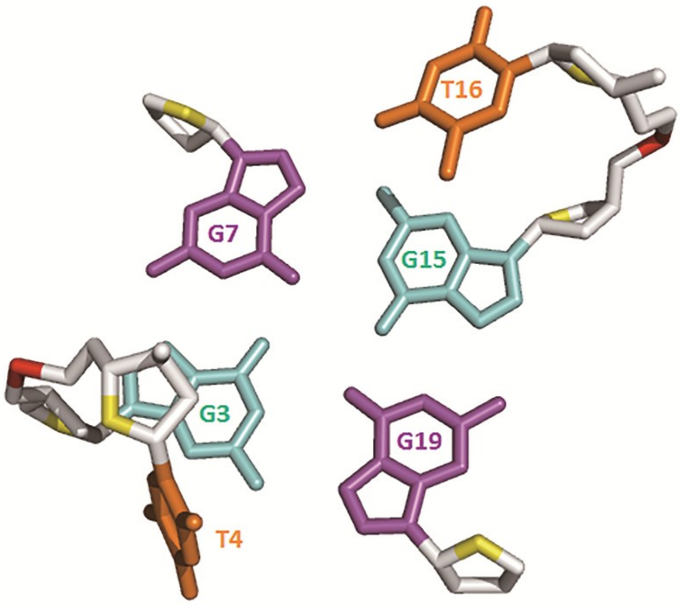

Figure S9. (a) The imino region of 1D ^{1}H-NMR spectra of *htel21*T_{18} with the assignment of guanine bases in G-quartets being indicated over the reference spectrum on top. Guanine imino protons were assigned with 1D ^{15}N-filtered HSQC spectra of samples containing site-specific low-enrichment (2%) ^{15}N-labelled oligonucleotides at the indicated positions. Spectra were recorded at 800 MHz (1D ^{1}H-NMR spectrum and 1D ^{15}N-filtered HSQC spectra) at 10°C in 5% D2O, 70 mM KCl, 20 mM potassium phosphate buffer (pH 7.0). (b) The imino and H8 regions of 2D HMBC spectrum showing correlation between imino proton and H8 proton within guanosine bases of *htel21*T_{18} sequence. Inset: through-bond correlations between guanosine imino and H8 protons via ^{13}C (at 5-position) at natural abundance using long-range J couplings. 2D HMBC spectrum was recorded at 800 MHz, 10°C in 5% D2O, 70 mM KCl, 20 mM potassium phosphate buffer (pH 7.0)

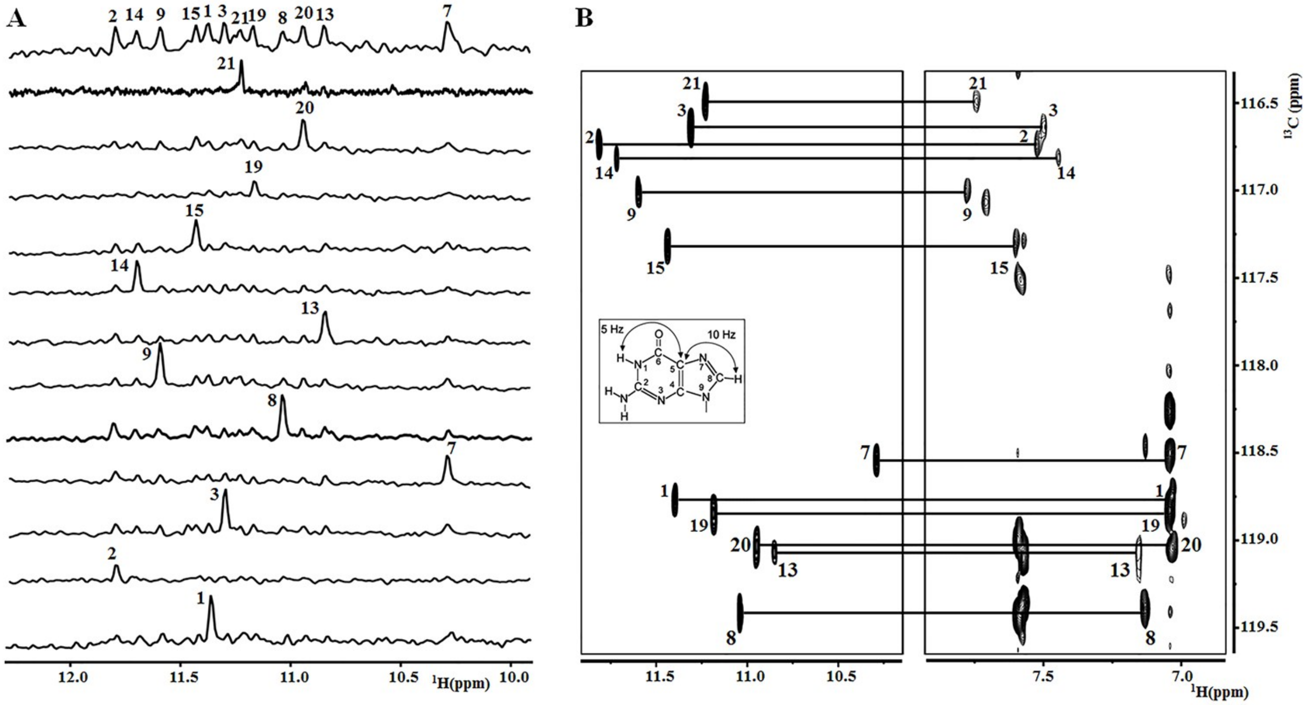

Figure S10 (a) The expanded H6-C6 regions of ^{13}C-^{1}H HSQC spectrum for T4, T5, T10, T11, T16, T17 and T18 of *htel21*T_{18}. Each sample contained site-specific low-enrichment (7%) ^{15}N, ^{13}C label at indicated positions 4, 5, 10, 11, 16, 17 and 18. **(b)** The expanded ribose H1'-C1' region of ^{13}C-^{1}H HSQC spectrum for T4, T5, T10, T11, T16, T17, T18 of *htel21*T_{18}. Each sample contained site-specific low-enrichment (7%) ^{15}N, ^{13}C label at indicated positions 4, 5, 10, 11, 16, 17 and 18. **(c)** Expanded H6-C6/H8-C8 region of ^{13}C-^{1}H HSQC spectrum of *htel21*T_{18}. **(d)** Expanded H2-C2 region of ^{13}C-^{1}H HSQC spectrum for A6 and A12 bases of *htel21*T_{18}.

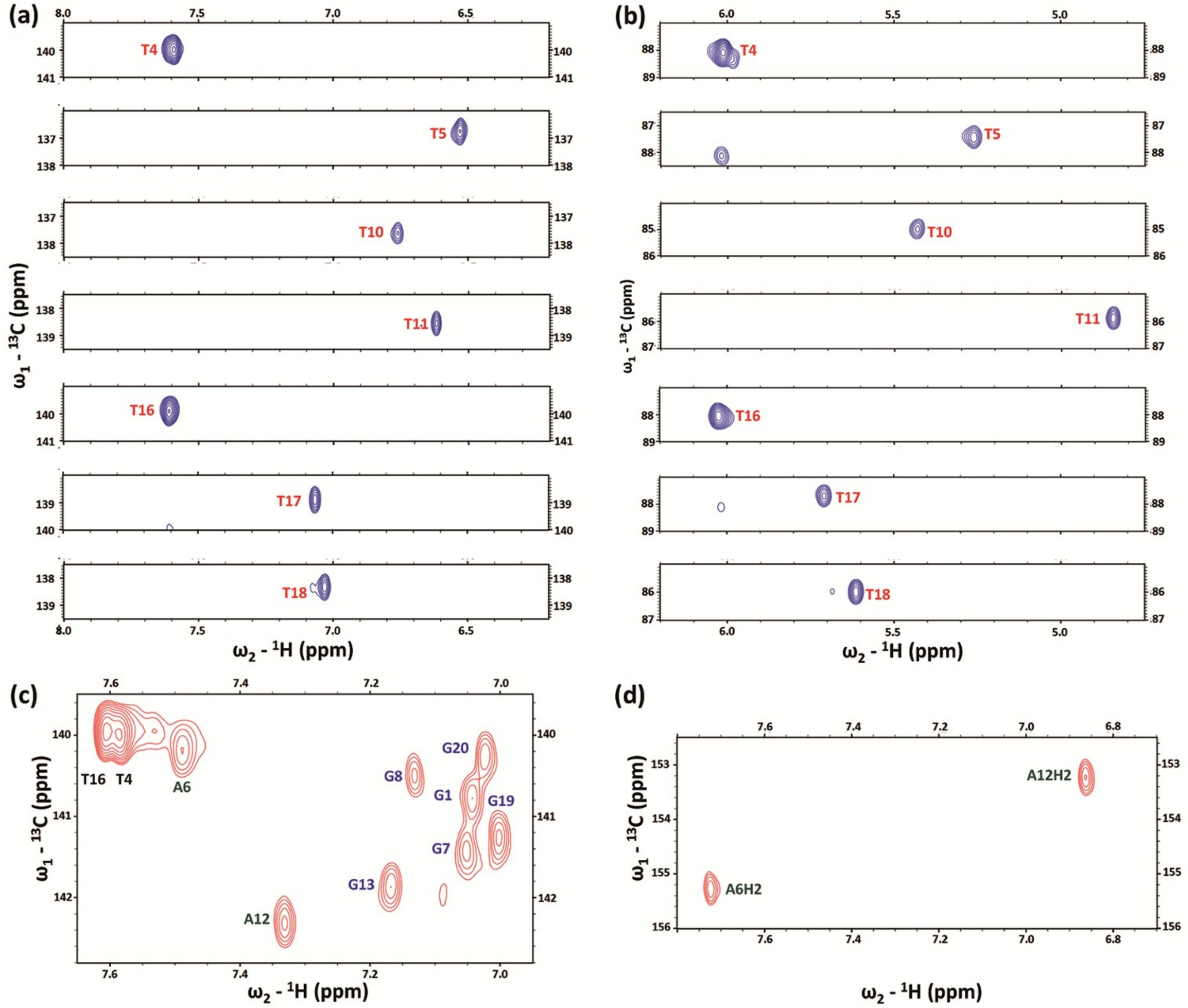

Figure S11 Expanded ^1H-^1H NOESY spectrum (75 ms mixing time) correlating base H8 and sugar H1' protons of *htel21*T_{18}. For *syn-anti* connection, both the *syn*G(i)H1'/*anti*G(i+1)H8 and *syn*G(i)H8/*anti*G(i+1)H1' NOEs are to be observed and only *syn*G(*i*)H8/*syn*G(*i*+1)H1' NOE can be observed for *syn-syn* connection.[1] The characteristic *syn*G1H8/*anti*G2H1' NOE and *syn*G19H8/*syn*G20H1', *syn*G7H8/*syn*G8H1' were shown in blue box. The characteristic *syn*G1H1'/*anti*G2H18 NOE were shown in black box. The missing T11 is indicated by blue star.

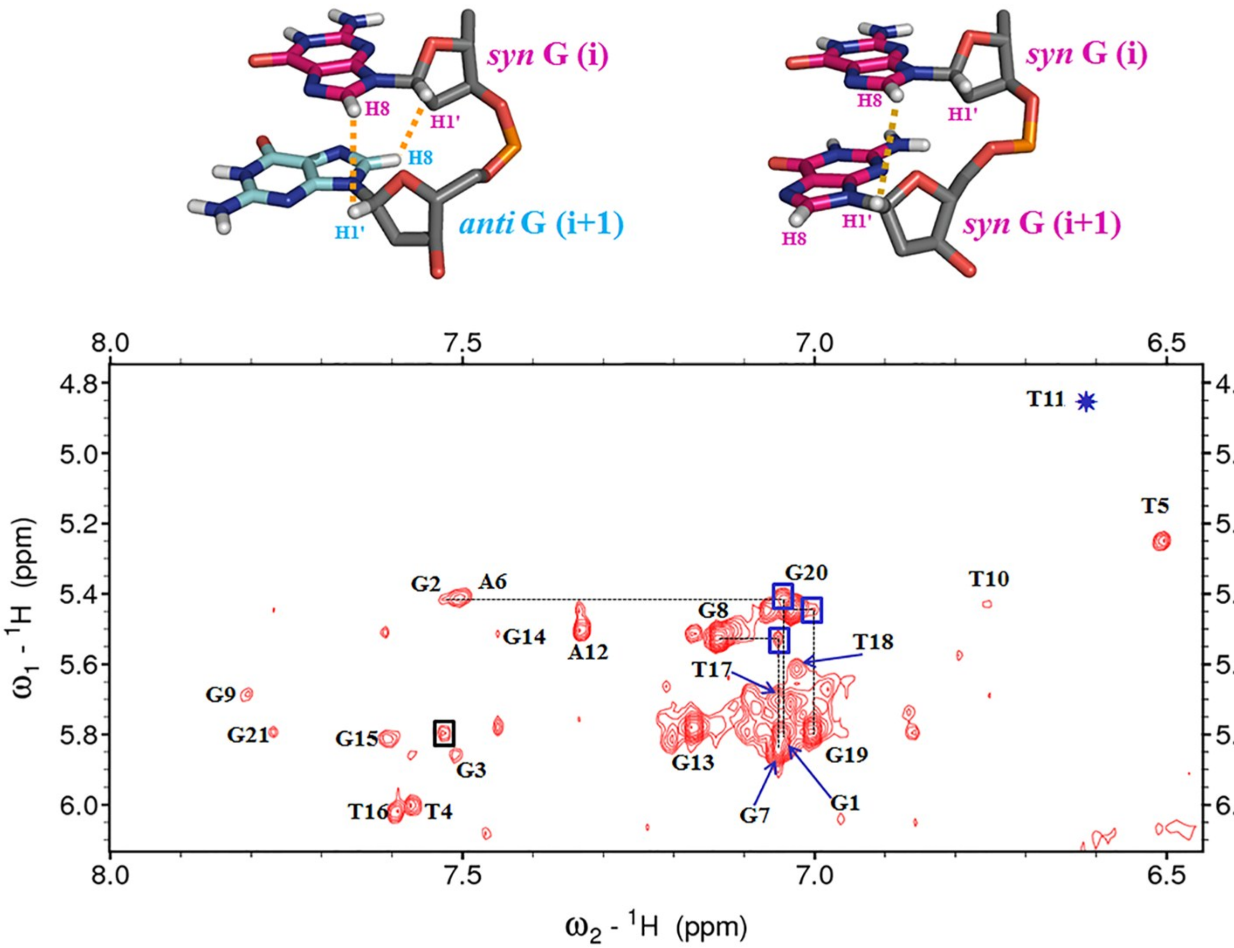

Table S1 Localization of *htel21*T_{18}, d[(GGGTTA)$_2$GGGTT*T*GGG], in the human genome. The result of sequence alignment was generated using the BLAT tool (http://genome.ucsc.edu/cgi-bin/hgBlat) from the UCSC Genome Browser database, the latest GRCh38/hg38 human assembly. There are a number of places where the sequence, d[(GGGTTA)$_2$GGGTT*T*GGG], occurs in the human genome such as chromosome 5, 8, 11, 17 and 19. The schematic view of the localization is shown. In each panel, the chromosome ID, architecture and mapping position (on a single-nucleotide scale) of *htel21*T_{18} are displayed. The *htel21*T_{18} sequence, d[(GGGTTA)$_2$GGGTT*T*GGG], is shown as red rectangle and the *htel21*, d[(GGGTTA)$_3$GGG],sequence is shown as blue rectangle. The positions of *htel21*T_{18} in the chromosome architecture are indicated by the red arrow.

Species (Latin name)	Species	Fragment size	Identity	Chromosome	Start position	End position
Homo sapiens	Human	21	100%	5	83516126	83516146
		21	100%	8	205311	205331
		21	100%	11	175332	175352
		21	100%	11	175440	175460
		21	100%	11	175641	175661
		21	100%	17	113145	113165
		21	100%	19	245590	245610

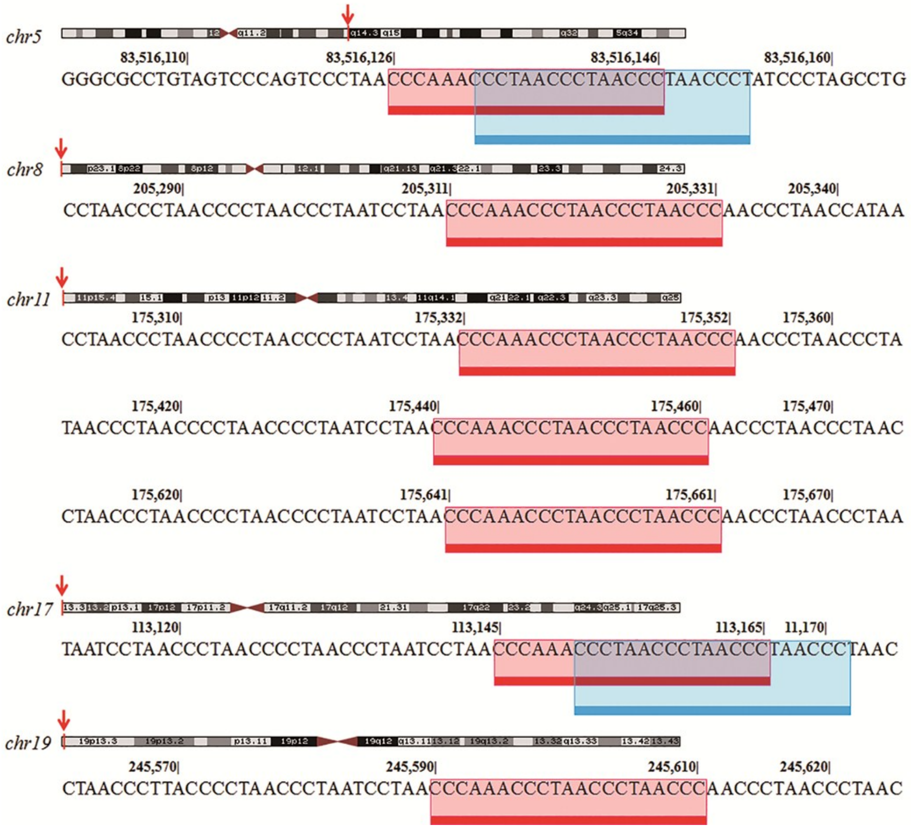

Table S2 Statistics of the calculated structures of a human telomeric variant DNA sequence,

d[$(GGGTTA)_2$GGGT*T*TGGG] named as *htel21*T_{18}, substituting an adenine A18 with thymine.

**NMR distance and dihedral constraints**	
**Distance Restraints**	
**Total NOE**	**356**
**Intraresidue distance restraints**	**126**
**Sequential (i, i + 1) distance restraints**	**121**
**Long-range (i, ≥ i + 2) distance restraints**	**57**
**Hydrogen bond restraints**	**52**
**Total dihedral angle restraints**	**62**
**Statistics for structures**	
**Violations (mean and SD)**	
**Number of NOE violations > 0.2Å**	**0 ± 0**
**R.m.s. deviation (Å) from experimental distance restraints**	**0.017 ± 0.001**
**Number of dihedral angle constraint violations > 5°**	**0 ± 0**
**R.m.s. deviation (°) from experimental dihedral angle restraints**	**0.553 ± 0.078**
**Deviations from idealized geometry**	
**Bond lengths (Å)**	**0.004 ± 0.0001**
**Bond angles (°)**	**0.470 ± 0.007**
**Improper (°)**	**0.337 ± 0.005**
**Pairwise all heavy atom RMSD values (Å)**	
**Three G-tetrads**	**0.42 ± 0.13**
**All residues**	**0.49 ± 0.12**

Table S3 Proton chemical shifts of *htel21*T_{18}, d[$(GGGTTA)_2$GGGTT*T*GGG].

	H1	H1'	H2	H2'	H2''	H21	H22	H3	H3'	H4'	H5#	H5'	H5''	H6	H61	H8
**G1**	**11.4**	**5.799**	**-**	**2.195**	**2.852**	**-**	**-**	**-**	**4.181**	**-**	**-**	**-**	**-**	**-**	**-**	**7.046**
**G2**	**11.83**	**5.417**	**-**	**2.199**	**2.858**	**9.786**	**5.717**	**-**	**-**	**-**	**-**	**-**	**-**	**-**	**-**	**7.525**
**G3**	**11.32**	**5.859**	**-**	**1.701**	**2.315**	**-**	**-**	**-**	**-**	**-**	**-**	**-**	**-**	**-**	**-**	**7.51**
**T4**	**-**	**6.005**	**-**	**1.866**	**2.147**	**-**	**-**	**-**	**4.473**	**4.037**	**1.694**	**3.9**	**3.755**	**7.574**	**-**	**-**
**T5**	**-**	**5.25**	**-**	**0.5599**	**1.48**	**-**	**-**	**9.595**	**4.293**	**-**	**0.9762**	**3.548**	**3.471**	**6.506**	**-**	**-**
**A6**	**-**	**5.406**	**7.715**	**2.273**	**2.461**	**-**	**-**	**-**	**-**	**-**	**-**	**3.64**	**3.394**	**-**	**6.293**	**7.497**
**G7**	**10.3**	**5.842**	**-**	**2.798**	**3.159**	**7.687**	**6.715**	**-**	**-**	**-**	**-**	**-**	**-**	**-**	**-**	**7.05**
**G8**	**11.05**	**5.522**	**-**	**2.106**	**2.39**	**8.931**	**5.788**	**-**	**-**	**-**	**-**	**4.139**	**3.943**	**-**	**-**	**7.136**
**G9**	**11.62**	**5.686**	**-**	**2.228**	**2.422**	**-**	**-**	**-**	**-**	**-**	**-**	**3.856**	**-**	**-**	**-**	**7.806**
**T10**	**-**	**5.434**	**-**	**1.649**	**1.893**	**-**	**-**	**-**	**4.39**	**3.777**	**1.299**	**3.926**	**3.753**	**6.759**	**-**	**-**
**T11**	**-**	**4.859**	**-**	**1.324**	**1.709**	**-**	**-**	**-**	**3.929**	**3.464**	**1.116**	**3.72**	**3.35**	**6.619**	**-**	**-**
**A12**	**-**	**5.503**	**6.861**	**1.805**	**2.328**	**-**	**-**	**-**	**-**	**-**	**-**	**3.403**	**2.986**	**-**	**-**	**7.332**
**G13**	**10.86**	**5.784**	**-**	**2.713**	**3.143**	**-**	**-**	**-**	**-**	**2.99**	**-**	**3.805**	**3.461**	**-**	**-**	**7.173**
**G14**	**11.73**	**5.511**	**-**	**2.066**	**2.391**	**9.737**	**5.786**	**-**	**-**	**-**	**-**	**3.999**	**3.921**	**-**	**-**	**7.449**
**G15**	**11.46**	**5.808**	**-**	**2.039**	**2.389**	**-**	**-**	**-**	**4.833**	**-**	**-**	**-**	**-**	**-**	**-**	**7.609**
**T16**	**-**	**6.019**	**-**	**1.764**	**2.23**	**-**	**-**	**-**	**4.446**	**4.083**	**1.751**	**3.877**	**3.765**	**7.598**	**-**	**-**
**T17**	**-**	**5.699**	**-**	**1.354**	**1.75**	**-**	**-**	**10.26**	**4.492**	**3.897**	**1.222**	**3.664**	**3.63**	**7.055**	**-**	**-**
**T18**	**-**	**5.613**	**-**	**2.083**	**2.198**	**-**	**-**	**13.35**	**-**	**3.823**	**0.9226**	**3.683**	**3.61**	**7.024**	**-**	**-**
**G19**	**11.2**	**5.789**	**-**	**2.841**	**3.204**	**-**	**-**	**-**	**-**	**-**	**-**	**-**	**4.079**	**-**	**-**	**7.005**
**G20**	**10.96**	**5.443**	**-**	**2.01**	**2.119**	**9.151**	**5.811**	**-**	**-**	**-**	**-**	**-**	**4.167**	**-**	**-**	**7.032**
**G21**	**11.26**	**5.795**	**-**	**2.058**	**2.312**	**-**	**-**	**-**	**-**	**-**	**-**	**3.792**	**-**	**-**	**-**	**7.769**

References:

1. M. Adrian, B. Heddi and A. T. Phan, *Methods*, 2012, **57**, 11-24.
2. R. V. Reshetnikov, A. M. Kopylov and A. V. Golovin, *Acta Naturae*, 2010, **2**, 72-81.
3. K. Padmanabhan, K. P. Padmanabhan, J. D. Ferrara, J. E. Sadler and A. Tulinsky, *Journal of Biological Chemistry*, 1993, **268**, 17651-17654.
4. K. W. Lim, P. Alberti, A. Guedin, L. Lacroix, J. F. Riou, N. J. Royle, J. L. Mergny and A. T. Phan, *Nucleic acids research*, 2009, **37**, 6239-6248.
5. Y. Xu, Y. Noguchi and H. Sugiyama, *Bioorganic & medicinal chemistry*, 2006, **14**, 5584-5591.
6. N. M. Smith, S. Amrane, F. Rosu, V. Gabelica and J. L. Mergny, *Chem Commun*, 2012, **48**, 11464-11466.
7. S. Amrane, R. W. L. Ang, Z. M. Tan, C. Li, J. K. C. Lim, J. M. W. Lim, K. W. Lim and A. T. Phan, *Nucleic acids research*, 2009, **37**, 931-938.
8. A. T. Phan, V. Kuryavyi, J. B. Ma, A. Faure, M. L. Andreola and D. J. Patel, *Proceedings of the National Academy of Sciences of the United States of America*, 2005, **102**, 634-639.
9. A. T. Phan, V. Kuryavyi, K. N. Luu and D. J. Patel, *Nucleic acids research*, 2007, **35**, 6517-6525.
